# Supplementary material for: Proton-driven alternating access in a spinster lipid transporter
Source: Nat Commun. 2022 Sep 2;13:5161. doi: 10.1038/s41467-022-32759-2 (PMC9440201; doi:10.1038/s41467-022-32759-2)
Supplement: Supplementary file 1 — Supplementary Information [file 41467_2022_32759_MOESM1_ESM.pdf]

Supplementary Information

Proton-driven alternating access in a spinster lipid transporter

Reza Dastvan[1,*], Ali Rasouli[2,3], Sepehr Dehghani-Ghahnaviyeh[2,3], Samantha Gies[1], Emad Tajkhorshid[2,*]

[1] Department of Biochemistry and Molecular Biology, Saint Louis University School of Medicine, St. Louis, MO 63104, USA

[2] Theoretical and Computational Biophysics Group, NIH Center for Macromolecular Modeling and Bioinformatics, Beckman Institute for Advanced Science and Technology, Department of Biochemistry, and Center for Biophysics and Quantitative Biology, University of Illinois at Urbana-Champaign, Urbana, IL 61801, USA

[3] These authors contributed equally to this work.

[*] To whom correspondence may be addressed. Email: reza.dastvan@health.slu.edu, emad@illinois.edu.

This PDF file includes:

Supplementary Figs. 1-34

Supplementary Tables 1-3

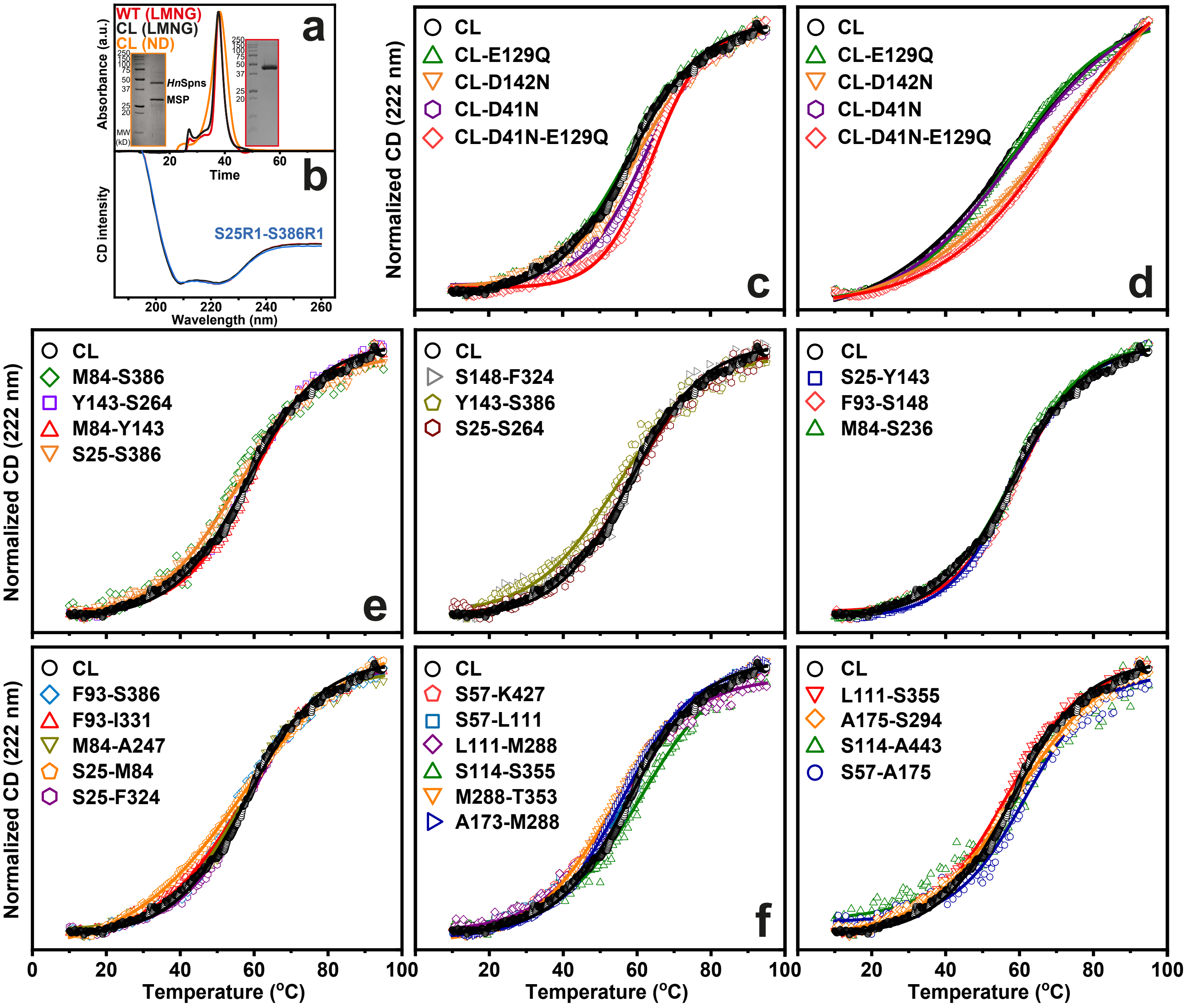

Supplementary Figure 1. Thermal stability analysis. (**a**) Size exclusion chromatography profiles of the wildtype (WT) and cysteine-less (CL) *Hn*Spns in LMNG micelles and nanodiscs (ND), and (**b**) their circular dichroism spectra in micelles along with a spin-labeled DEER mutant (S25R1-S386R1). (**c**) Thermal stability of the protonation-mimetic mutants in LMNG micelles and (**d**) in lipid nanodiscs, compared with the CL protein. (**e**) Thermal stability of the spin-labeled DEER mutants on the intracellular and (**f**) extracellular side in LMNG micelles compared with CL protein. n_{CL} = 3 and n_{mutants} = 1. Melting temperatures (T_m) are reported in Supplementary Table 1.

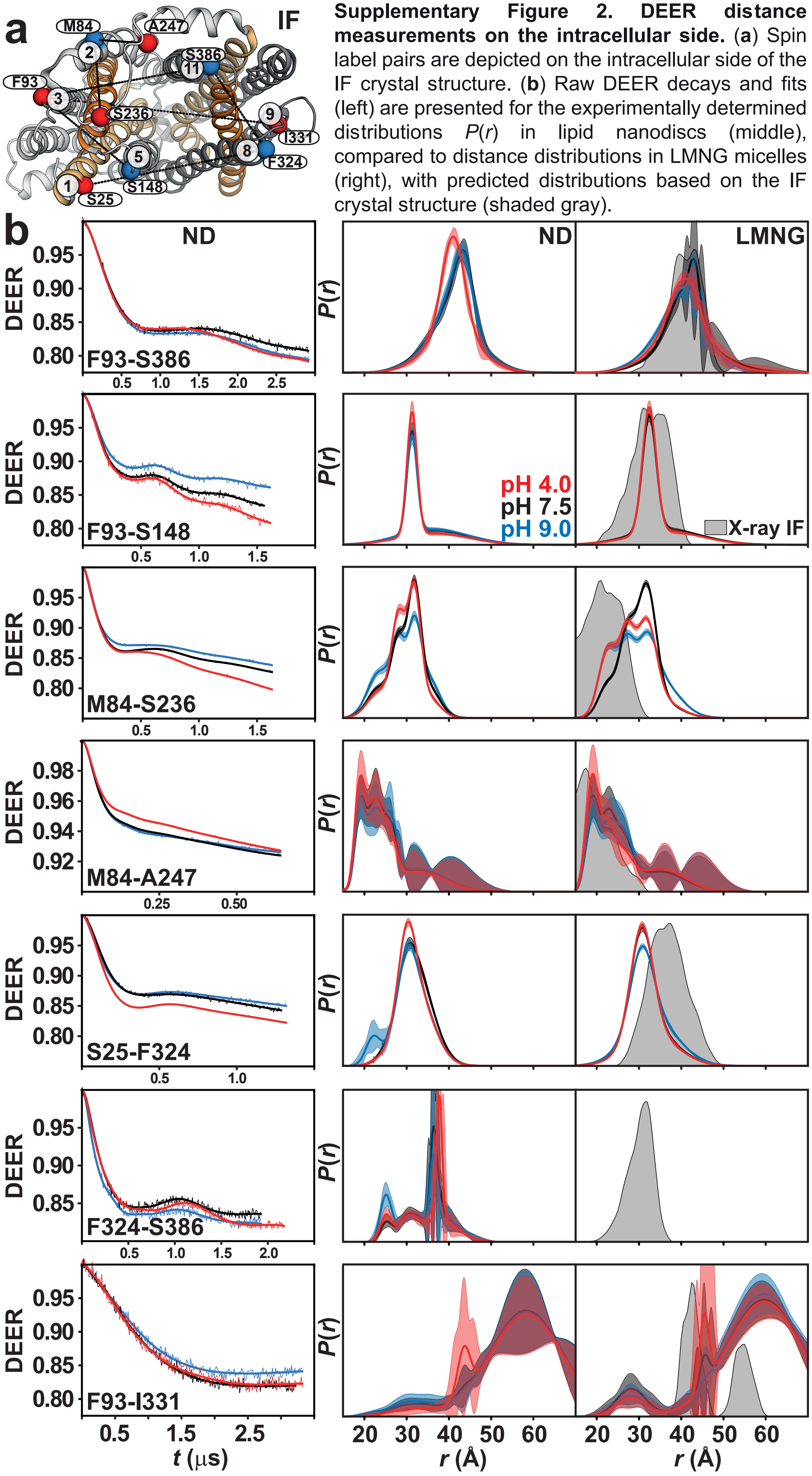

Supplementary Figure 2. DEER distance measurements on the intracellular side. (**a**) Spin label pairs are depicted on the intracellular side of the IF crystal structure. (**b**) Raw DEER decays and fits (left) are presented for the experimentally determined distributions *P*(*r*) in lipid nanodiscs (middle), compared to distance distributions in LMNG micelles (right), with predicted distributions based on the IF crystal structure (shaded gray).

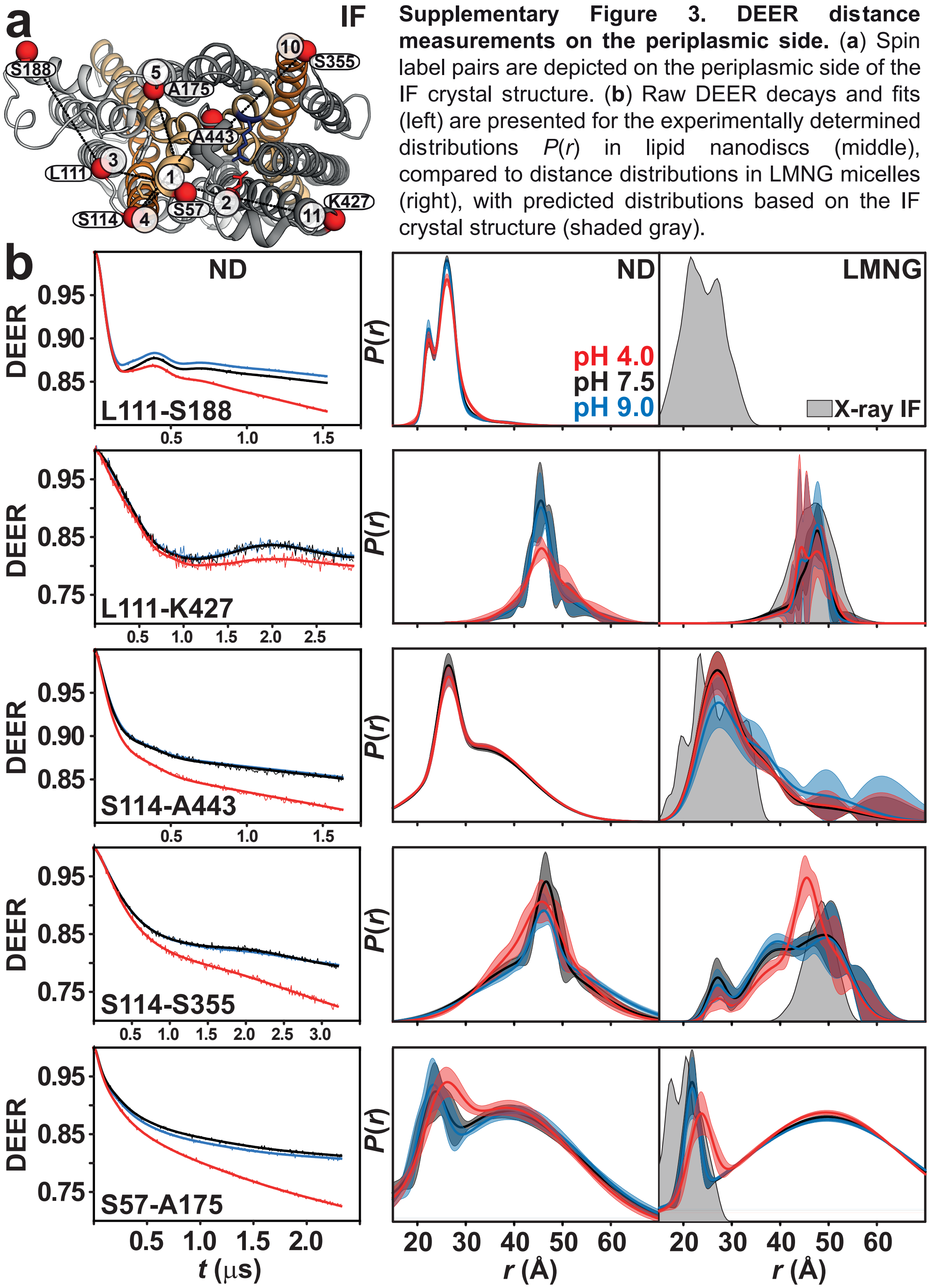

Supplementary Figure 3. DEER distance measurements on the periplasmic side. (**a**) Spin label pairs are depicted on the periplasmic side of the IF crystal structure. (**b**) Raw DEER decays and fits (left) are presented for the experimentally determined distributions *P*(*r*) in lipid nanodiscs (middle), compared to distance distributions in LMNG micelles (right), with predicted distributions based on the IF crystal structure (shaded gray).

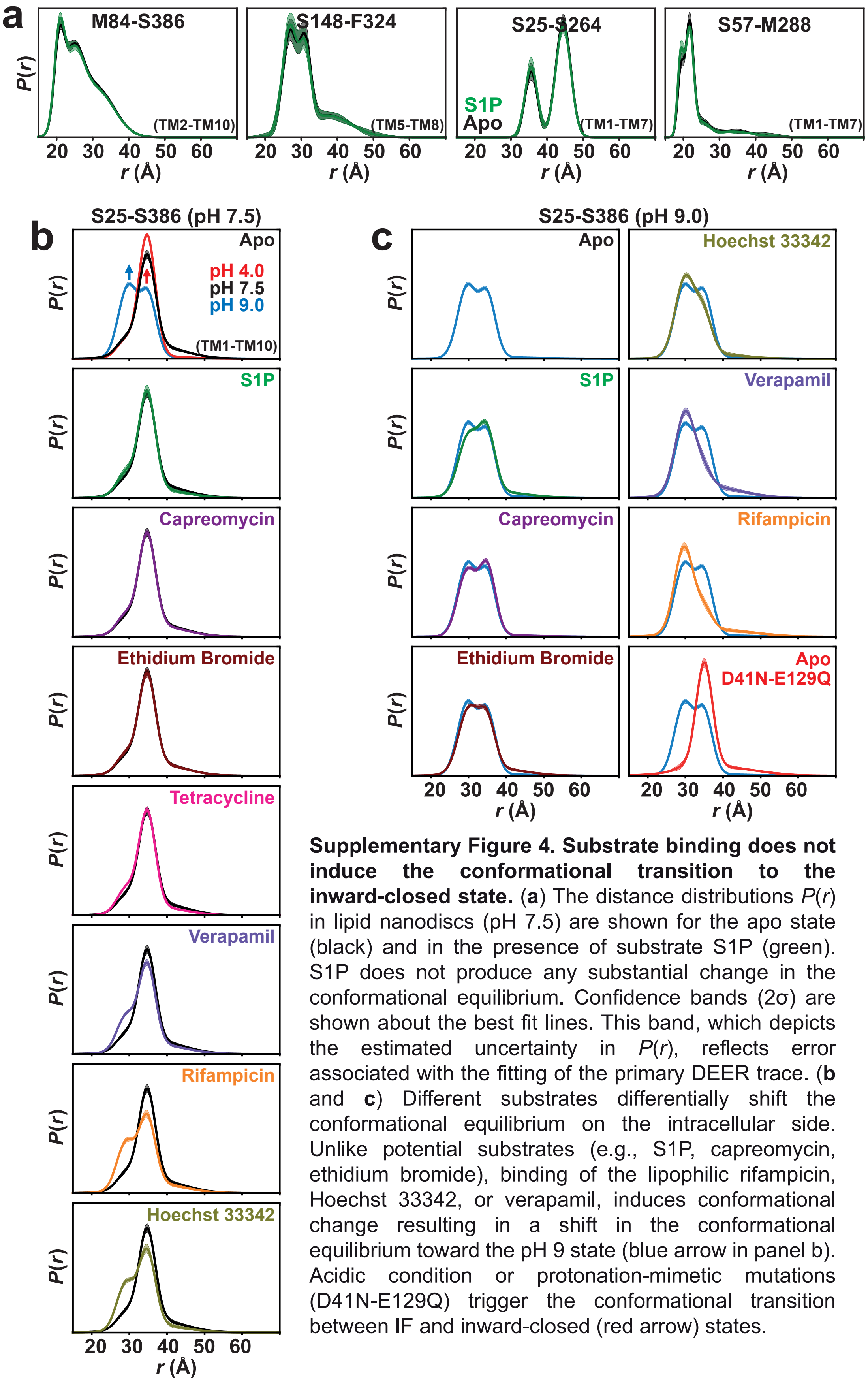

Supplementary Figure 4. Substrate binding does not induce the conformational transition to the inward-closed state. (**a**) The distance distributions *P*(*r*) in lipid nanodiscs (pH 7.5) are shown for the apo state (black) and in the presence of substrate S1P (green). S1P does not produce any substantial change in the conformational equilibrium. Confidence bands (2σ) are shown about the best fit lines. This band, which depicts the estimated uncertainty in *P*(*r*), reflects error associated with the fitting of the primary DEER trace. (**b** and **c**) Different substrates differentially shift the conformational equilibrium on the intracellular side. Unlike potential substrates (e.g., S1P, capreomycin, ethidium bromide), binding of the lipophilic rifampicin, Hoechst 33342, or verapamil, induces conformational change resulting in a shift in the conformational equilibrium toward the pH 9 state (blue arrow in panel b). Acidic condition or protonation-mimetic mutations (D41N-E129Q) trigger the conformational transition between IF and inward-closed (red arrow) states.

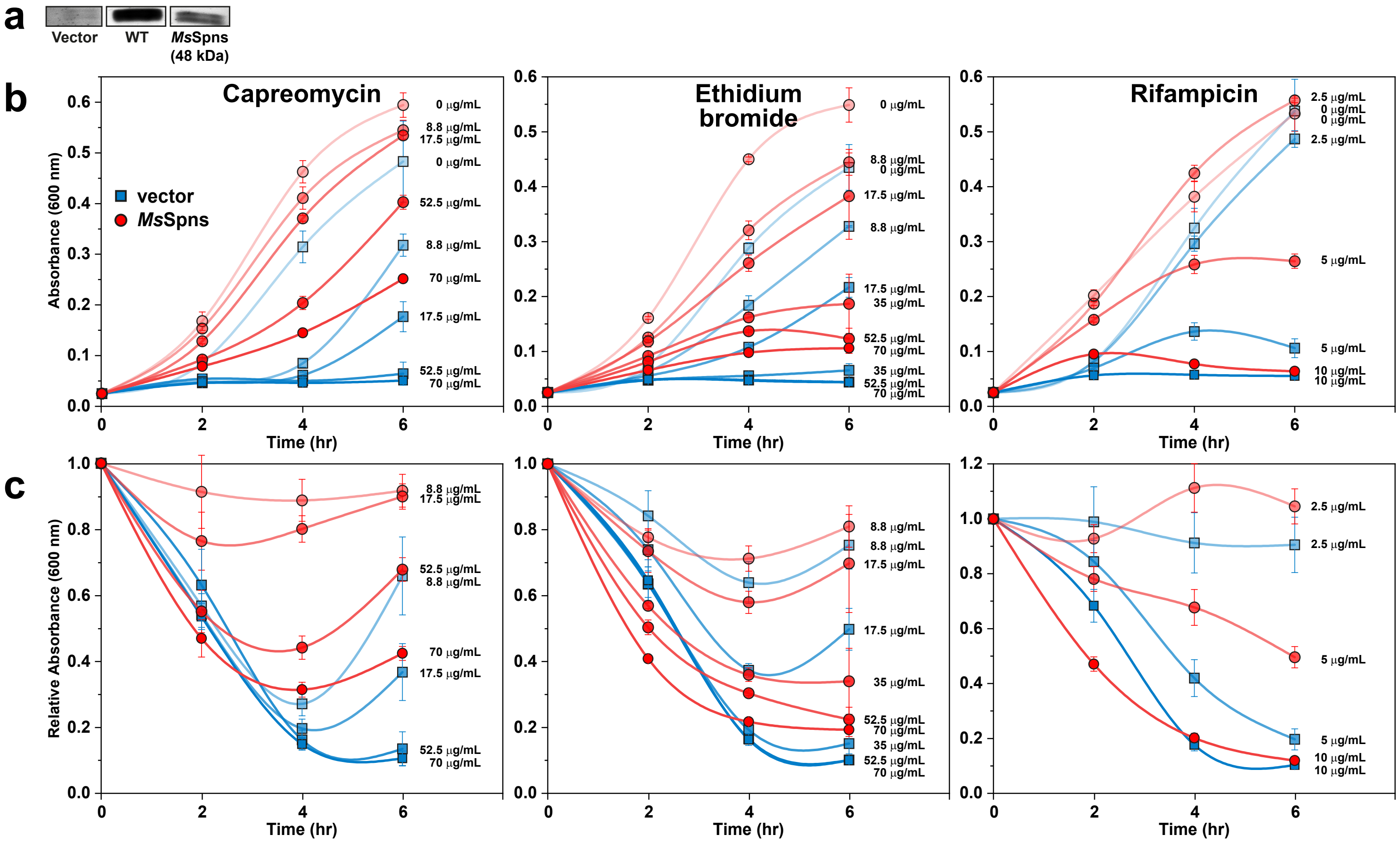

Supplementary Figure 5. Cell growth-based drug resistance assays on *Mycobacterium smegmatis* MDR efflux transporter MSMEG_3705 (*Ms*Spns). (**a**) The relative expression of WT *Ms*Spns in the cell growth assays was visualized by SDS-PAGE and staining with InVision His tag stain, confirming expression. Purified *Ms*Spns used as a standard is shown for comparison. (**b**) Cell growth for the WT protein relative to the vector at 37 °C, in the presence of different concentrations of capreomycin, ethidium bromide and rifampicin. (**c**) To account for the growth behavior of the vector and *Ms*Spns in the absence of drug, absorbances at 600 nm in the presence of drugs were divided by the 0 µg/mL drug well. Data are presented as mean values ± standard deviation. Each data point was performed in triplicate. The *p* values were determined by an unpaired *t* test and reported in Supplementary Tables 2 and 3. Source data is available as a Source Data file.

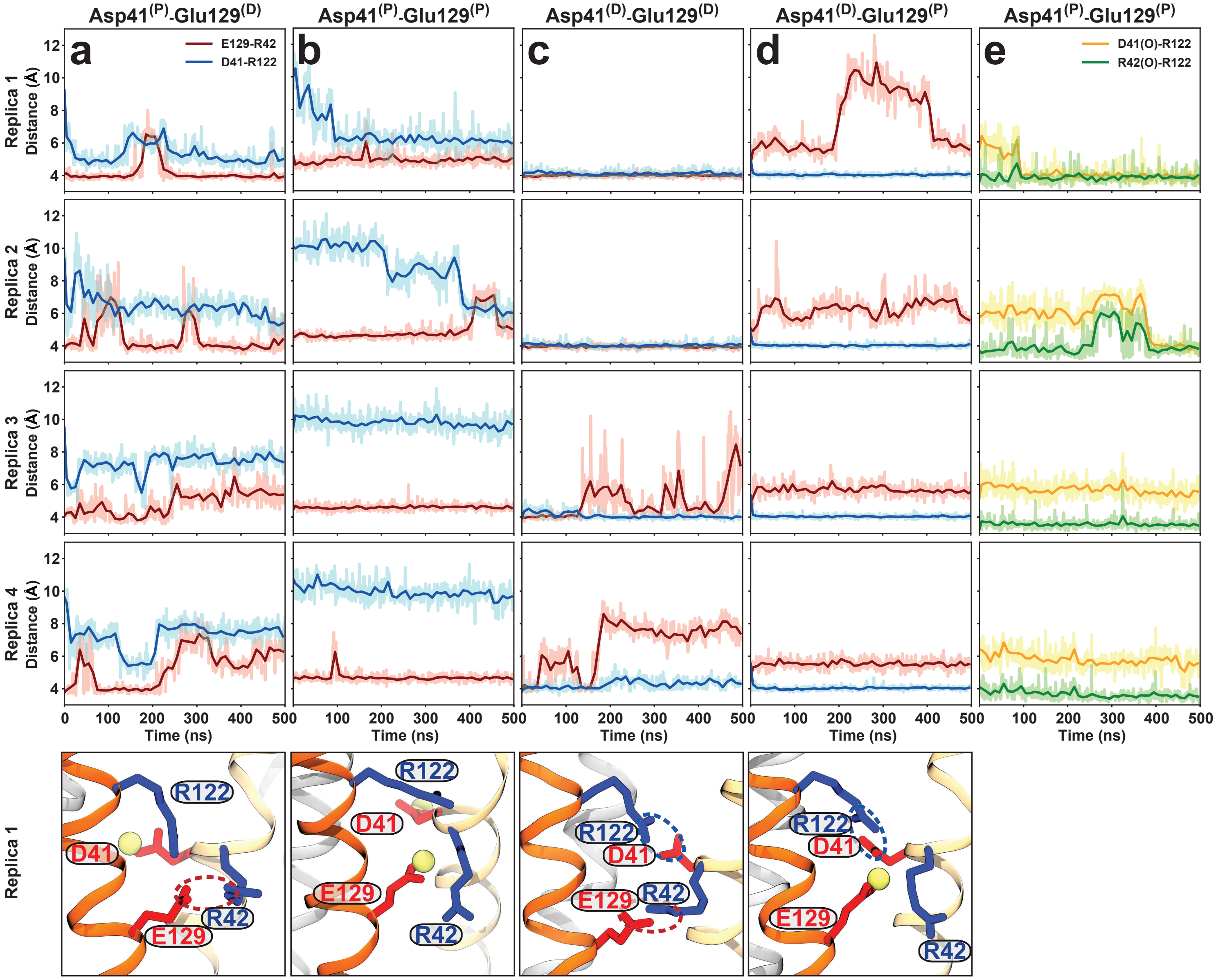

Supplementary Figure 6. Molecular dynamics simulations of IF *Hn*Spns in lipid membranes. (**a**-**d**) Different permutations of protonation states of Asp41 and Glu129 (indicated by D and P for deprotonated and protonated, respectively) and their interactions with two nearby basic residues (four different systems in four independent copies). Time series (light color) and their moving averages (dark color) of Glu129-Arg42 (red) and Asp41-Arg122 (blue) side-chain distances from MD simulations of the IF *Hn*Spns structure in membrane are used to monitor the stability of functionally relevant salt bridges in simulations under different titration states. (**e**) Double protonation of these acidic residues stabilizes a distinct conformation in which the side chain of Arg122 continuously interacts with the backbone oxygen of Arg42 (green time series) but only transiently with Asp41 (orange time series). Molecular snapshots (bottom) are captured from the last frame of each trajectory of the first replica. Acidic and basic residues are shown in red and blue, respectively, and the proton is represented with a yellow sphere.

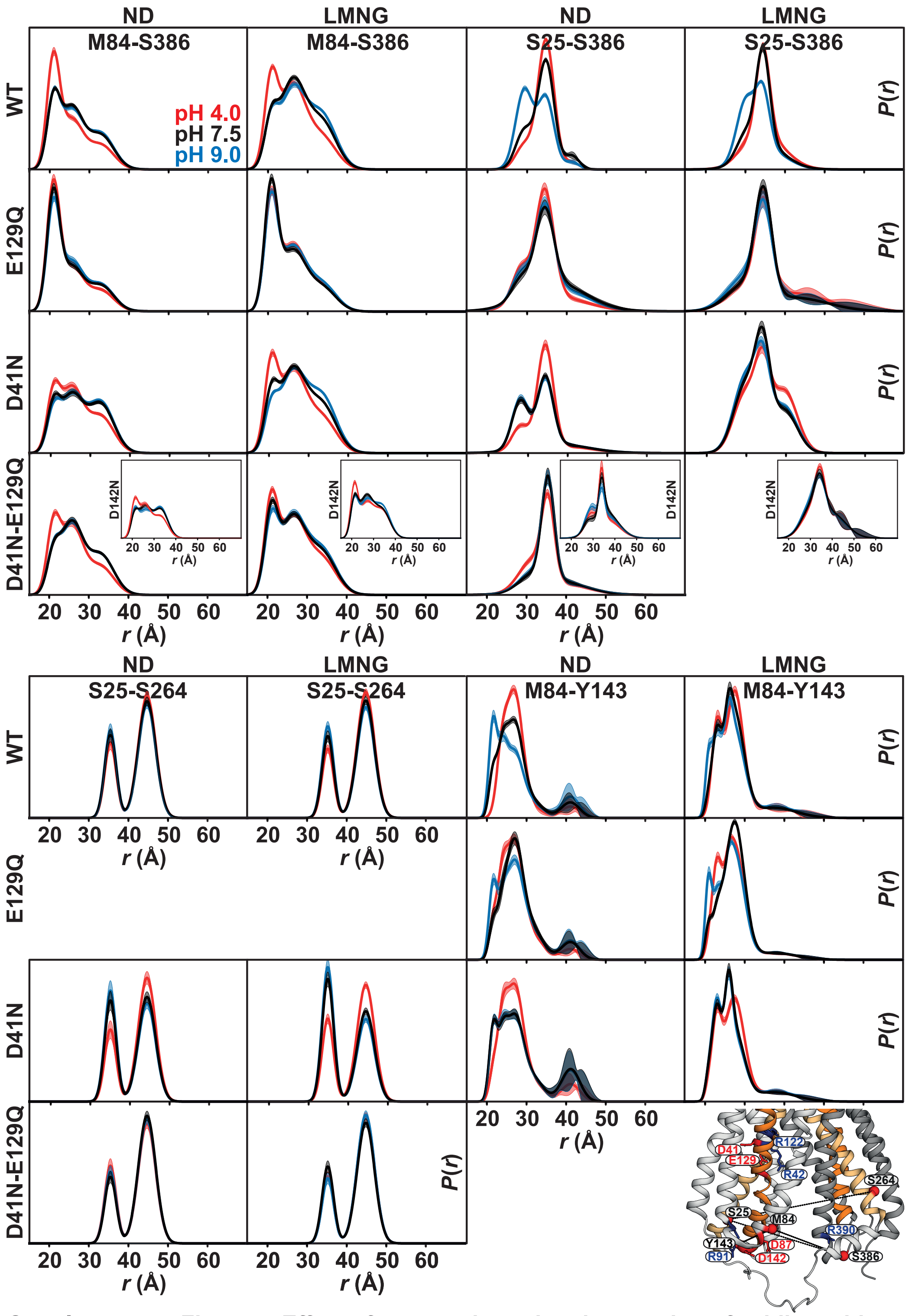

Supplementary Figure 7. Effect of protonation-mimetic mutation of acidic residues on conformational equilibrium on the intracellular side in lipid nanodiscs and detergent micelles. The single (E129Q, D41N, D142N) or double (D41N/E129Q) mutations were combined with the double-cysteine mutations. DEER experiments in the presence of these mutations support the results of the MD simulations and reveal that Asp41 protonation has the opposite effect from that of Glu129 on the conformational changes on the intracellular side. Confidence bands (2σ) are shown about the best fit lines. This band, which depicts the estimated uncertainty in $P(r)$, reflects error associated with the fitting of the primary DEER trace.

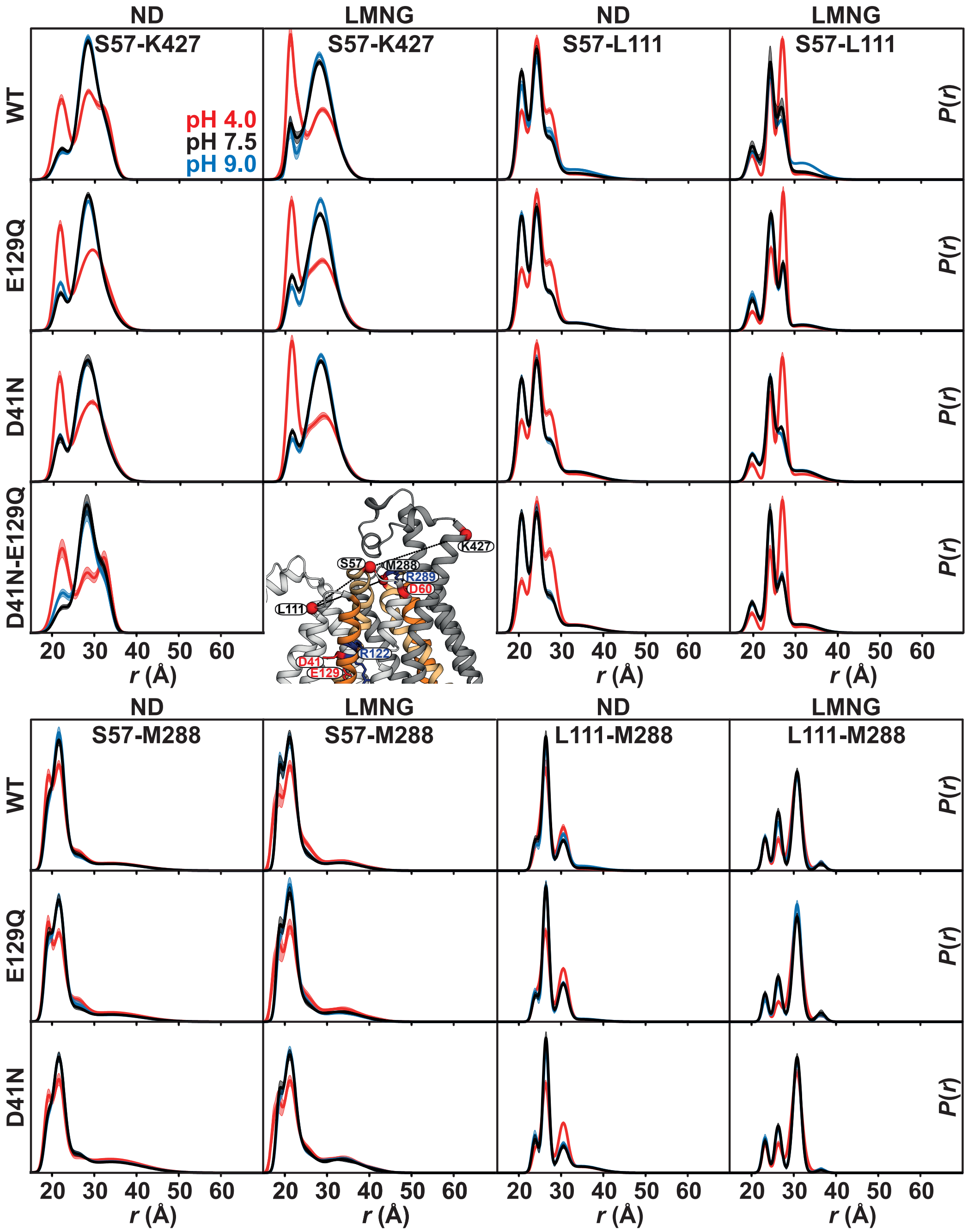

Supplementary Figure 8. Effect of protonation-mimetic mutation of acidic residues on conformational equilibrium on the periplasmic side. The single (E129Q, D41N, D142N) or double (D41N/E129Q) mutations were combined with the double-cysteine mutations. DEER experiments in the presence of these mutations reveal that the periplasmic side remains mostly invariant in response to these mutations. Confidence bands (2σ) are shown about the best fit lines. This band, which depicts the estimated uncertainty in *P*(*r*), reflects error associated with the fitting of the primary DEER trace.

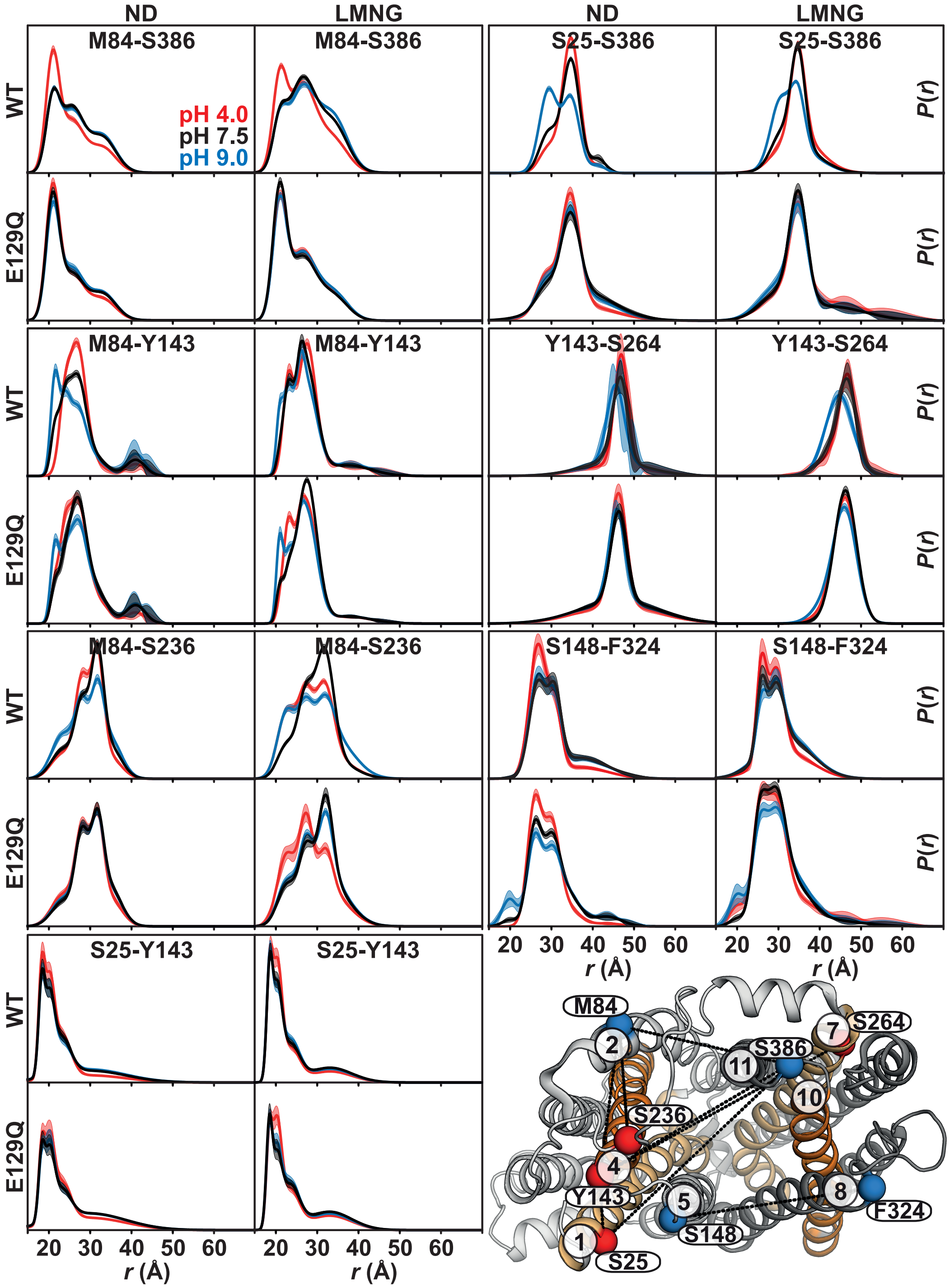

Supplementary Figure 9. Effect of protonation-mimetic mutation of the intracellular protonation master switch glutamate 129 (E129Q) on conformational equilibrium on the intracellular side. The E129Q mutation was combined with the double-cysteine mutations. The E129Q mutation captures the transporter in the pH 4 population state, i.e., the inward-closed conformation. Thus, our data suggest that E129 is the intracellular protonation master switch regulating the conformational transition between IF and inward-closed states. Confidence bands (2σ) are shown about the best fit lines.

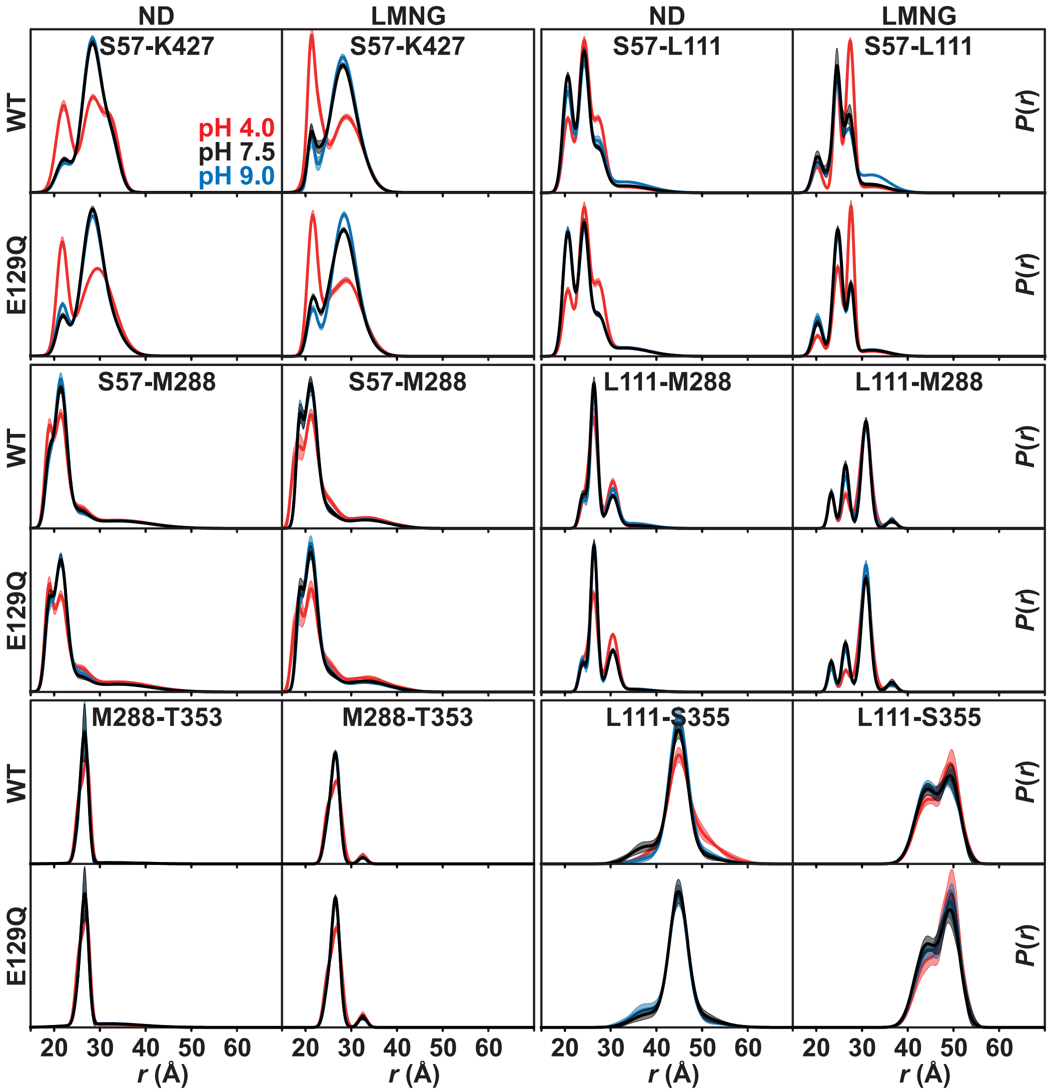

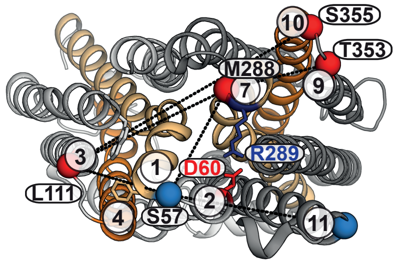

Supplementary Figure 10. Effect of protonation-mimetic mutation of the intracellular protonation master switch glutamate 129 (E129Q) on conformational equilibrium on the periplasmic side. The E129Q mutation was combined with the double-cysteine mutations. DEER experiments reveal that the periplasmic side remains mostly invariant in response to this mutation. Confidence bands (2σ) are shown about the best fit lines.

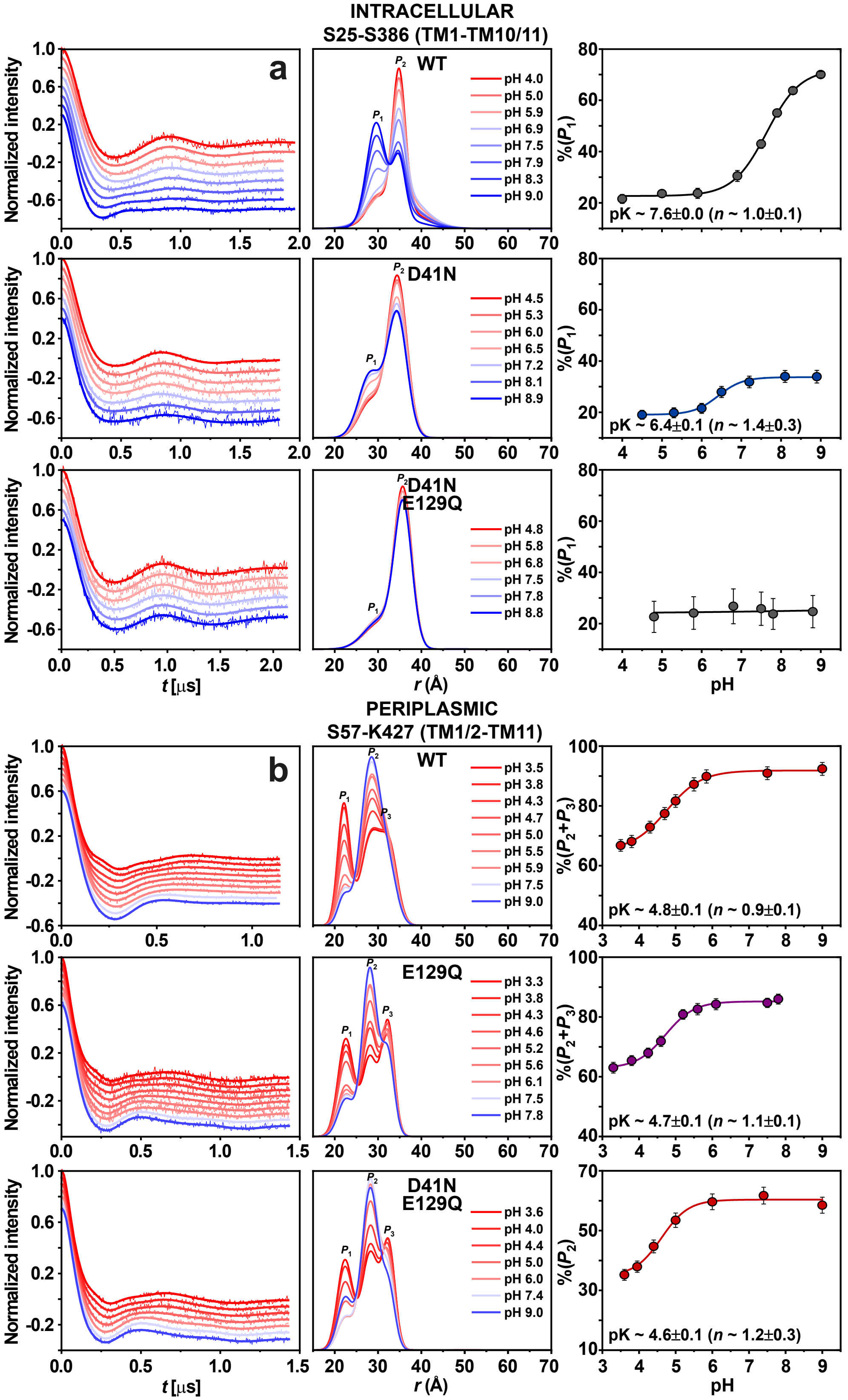

Supplementary Figure 11. Intracellular and periplasmic sides of *Hn*Spns sense its protonation state differently. (**a**) Distance distributions of intracellular (S25-S386) and (**b**) periplasmic (S57-K427) pairs were obtained at different pH values in lipid nanodiscs with and without protonation-mimetic mutations of acidic residues. The variation in population of rising or decreasing distance peaks as a function of pH was used to estimate the pK value for conformational changes in *Hn*Spns. The D41N-E129Q double mutation abrogates conformational changes on the intracellular side, with periplasmic side mainly unaffected, demonstrating these proton switches mainly control conformational changes on the intracellular side. From left to right, baseline-corrected and normalized DEER traces along with the fits, obtained distance distributions, and the variation in populations as a function of pH are shown. (right panels) The error bars for the populations of distance peaks (center: population from the best fit, middle panels) represent the uncertainty in the fit parameter reported at the 2σ (95%) confidence level. Source data are available as a Source Data file.

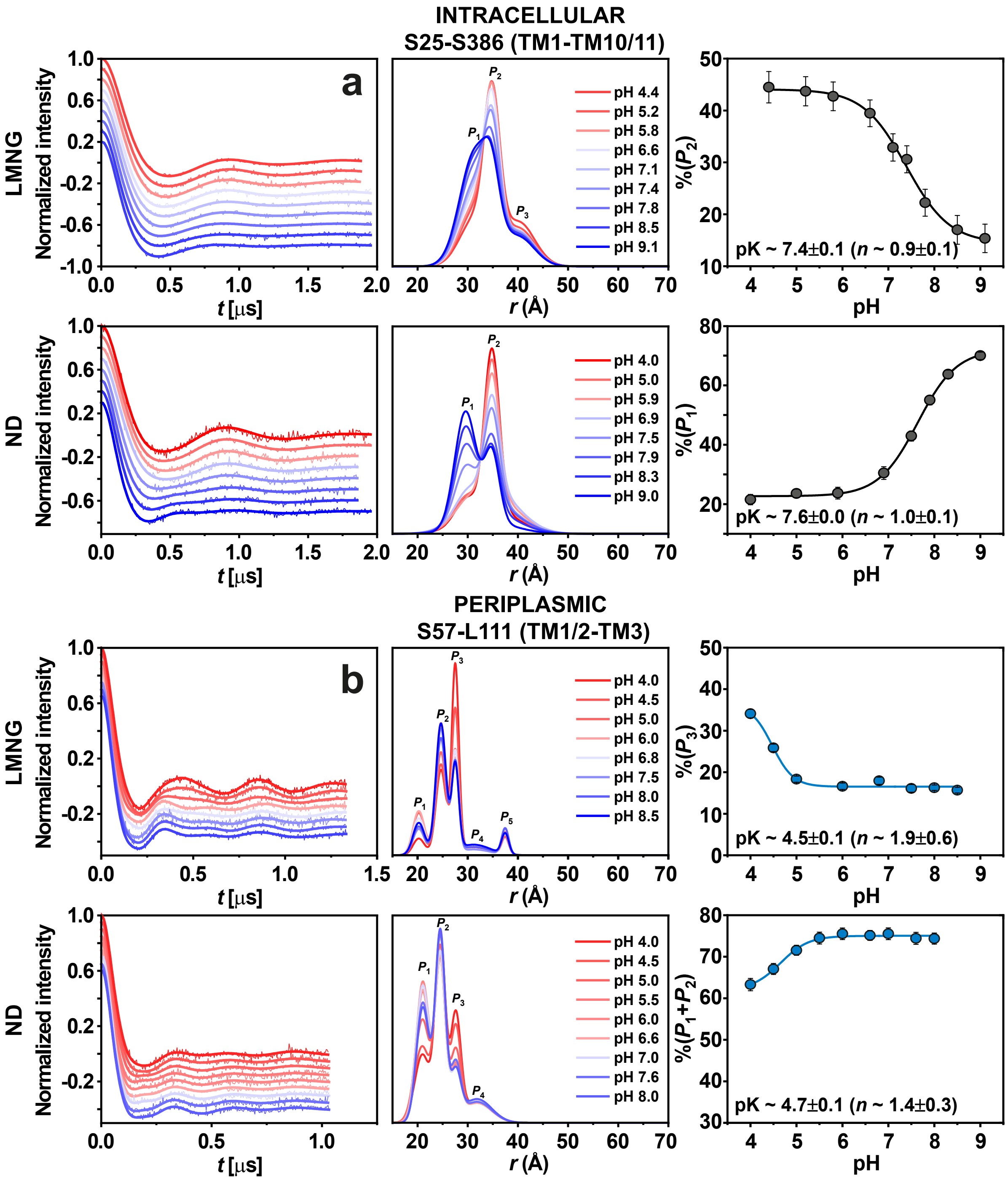

Supplementary Figure 12. Effect of environment (lipid nanodiscs vs. micelles) on sensing the protonation state. (**a**) Distance distributions of intracellular (S25-S386) and (**b**) periplasmic (S57-L111) pairs were obtained at different pH values in lipid nanodiscs and LMNG micelles. Similar pK values were obtained in LMNG micelles and lipid nanodiscs. From left to right, baseline-corrected and normalized DEER traces along with the fits, obtained distance distributions, and the variation in population of rising or decreasing distance peaks as a function of pH are shown and used to estimate the pK values for conformational changes in *Hn*Spns. (right panels) The error bars for the populations of distance peaks (center: population from the best fit, middle panels) represent the uncertainty in the fit parameter reported at the 2σ (95%) confidence level. Source data are available as a Source Data file.

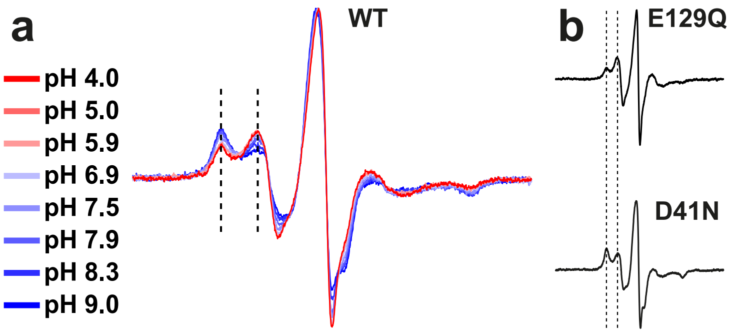

Supplementary Figure 13. The pH-dependence of the CW EPR spectra of (**a**) the S25-S386 (TM1-TM10/11) intracellular pair. (**b**) The D41N and E129Q mutations on the same cysteine pair shift the equilibrium in opposite directions towards basic and acidic pH states, respectively.

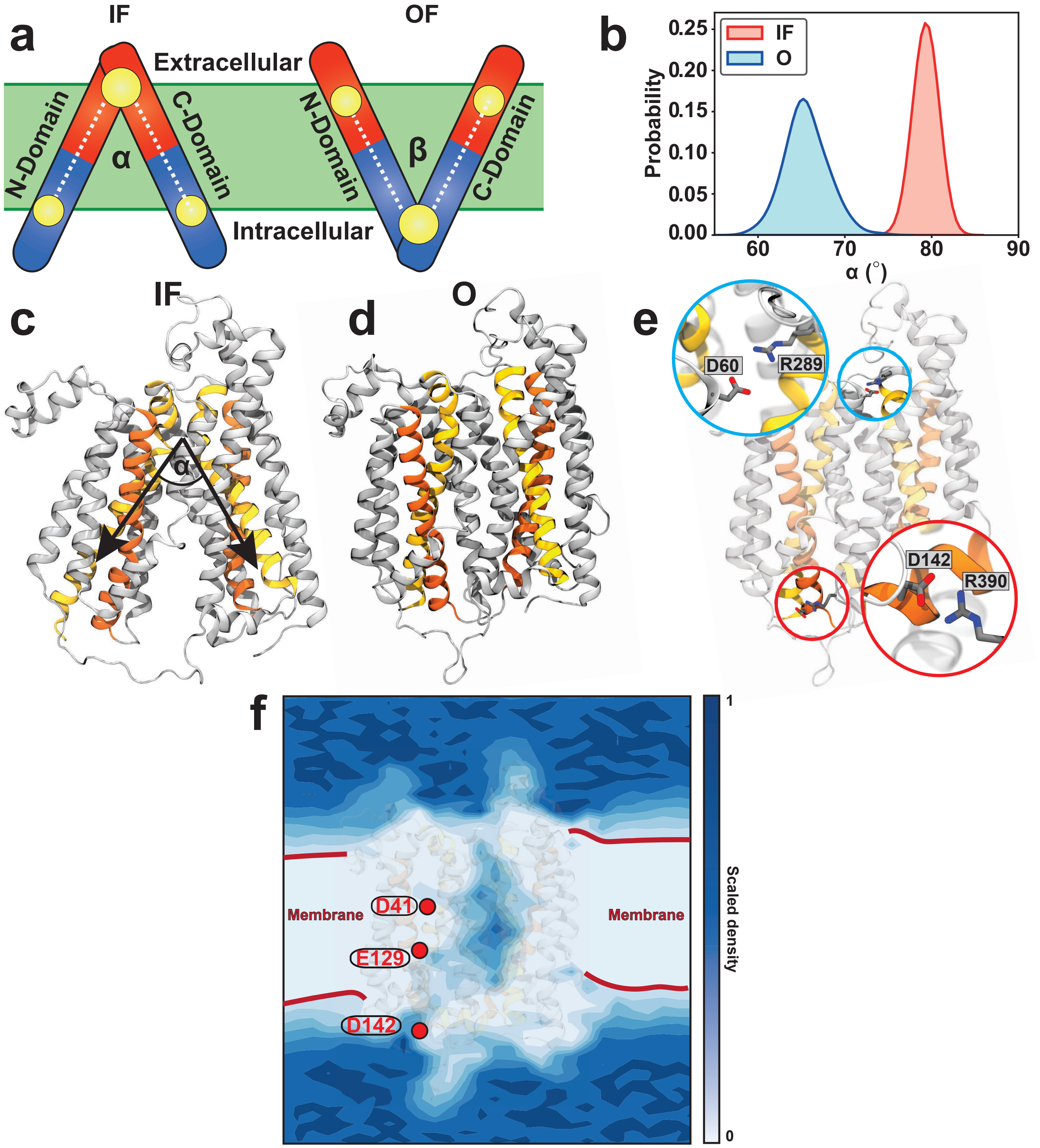

Supplementary Figure 14. Capturing a stable O conformation of *Hn*Spns. (**a**) Two orientation-based collective variables, α and β, representing the intracellular and extracellular opening/closing of the transporter, respectively, and used to control the conformation of the transporter. N- and C-domain are split into two equal segments shown in red and blue, representing the extracellular and intracellular halves, respectively. α is defined as the angle between the centers of masses of (1) the intracellular half of N-domain, (2) the extracellular half of the protein (including both N- and C-domains), and (3) the intracellular half of C-domain. β is the angle between the centers of masses of (1) the extracellular half of N-domain, (2) the intracellular half of the protein (including both N- and C-domains), and (3) the extracellular half of C-domain. (**b**) Probability distribution of the intracellular opening of the transporter for the IF and stabilized O conformations through 500 ns of equilibrium simulations. (**c**) The IF conformation and definition of the intracellular opening angle. (**d**) The stable O conformation. (**e**) Periplasmic (Arg289^{TM7}-Asp60^{TM2}) and intracellular (Arg390^{TM11}-Asp142^{TM4}) salt bridges are formed, stabilizing the O conformation. (**f**) 2D histogram of the calculated water density for the last 250 ns of the O state equilibrium trajectory.

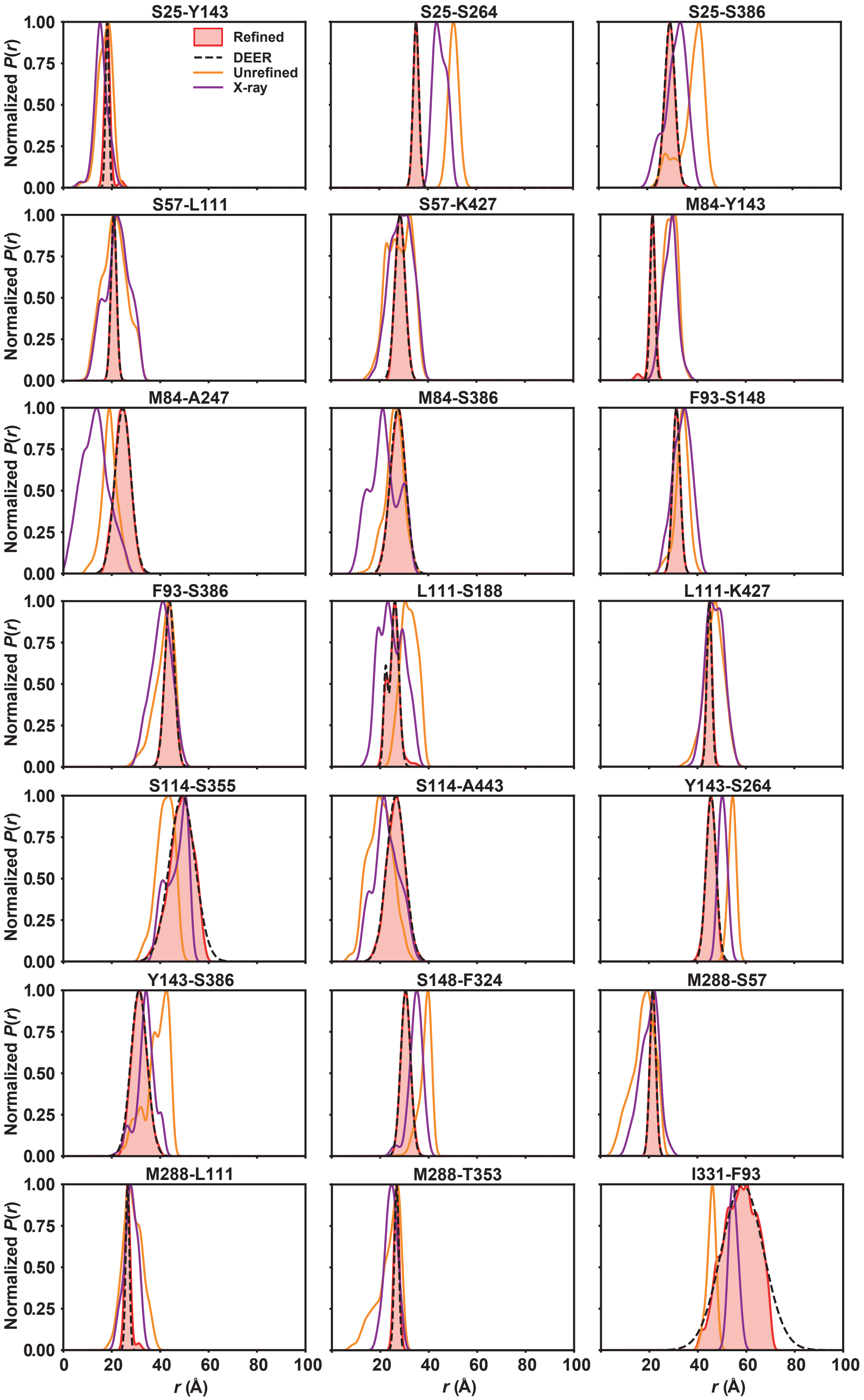

Supplementary Figure 15. DEER-guided refinement of IF *Hn*Spns structure in lipid membranes. Comparison of the distance distributions from the refined structure (shaded red) to the experimental pH 9 DEER distance histograms in nanodiscs (dashed black lines), the relaxed unrefined template (orange lines), and the predicted distributions on the crystal structure (purple lines). The refined model matches the experimental distance populations.

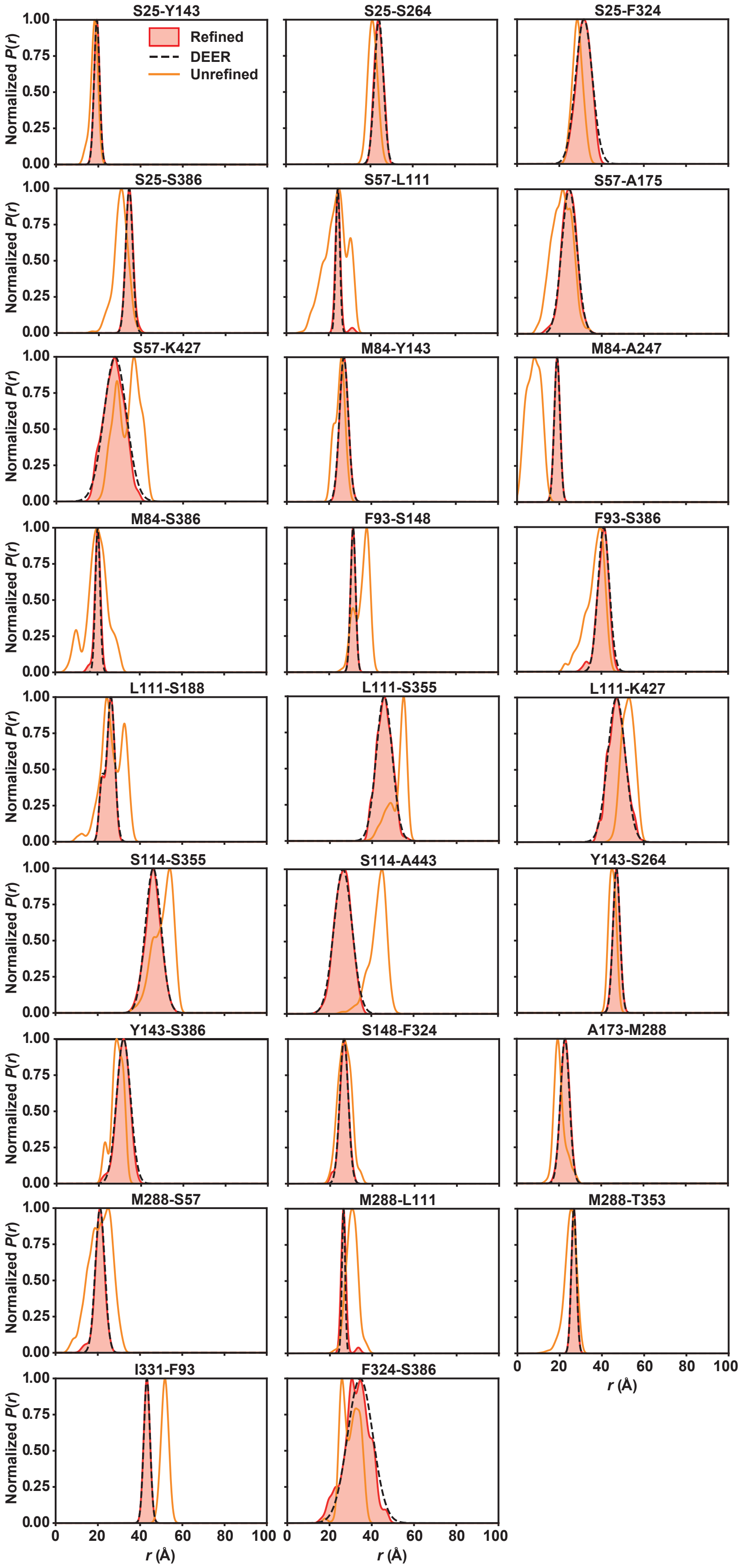

Supplementary Figure 16. Refinement of the O state of *Hn*Spns in lipid membrane. Comparison of the distance distributions from the refined model (shaded red) to the experimental pH 4 DEER distance histograms in nanodiscs (dashed black lines), and the unrefined MD-generated O conformation without imposing the harmonic restraints on the probes (orange lines). The refined model matches the experimental distance populations.

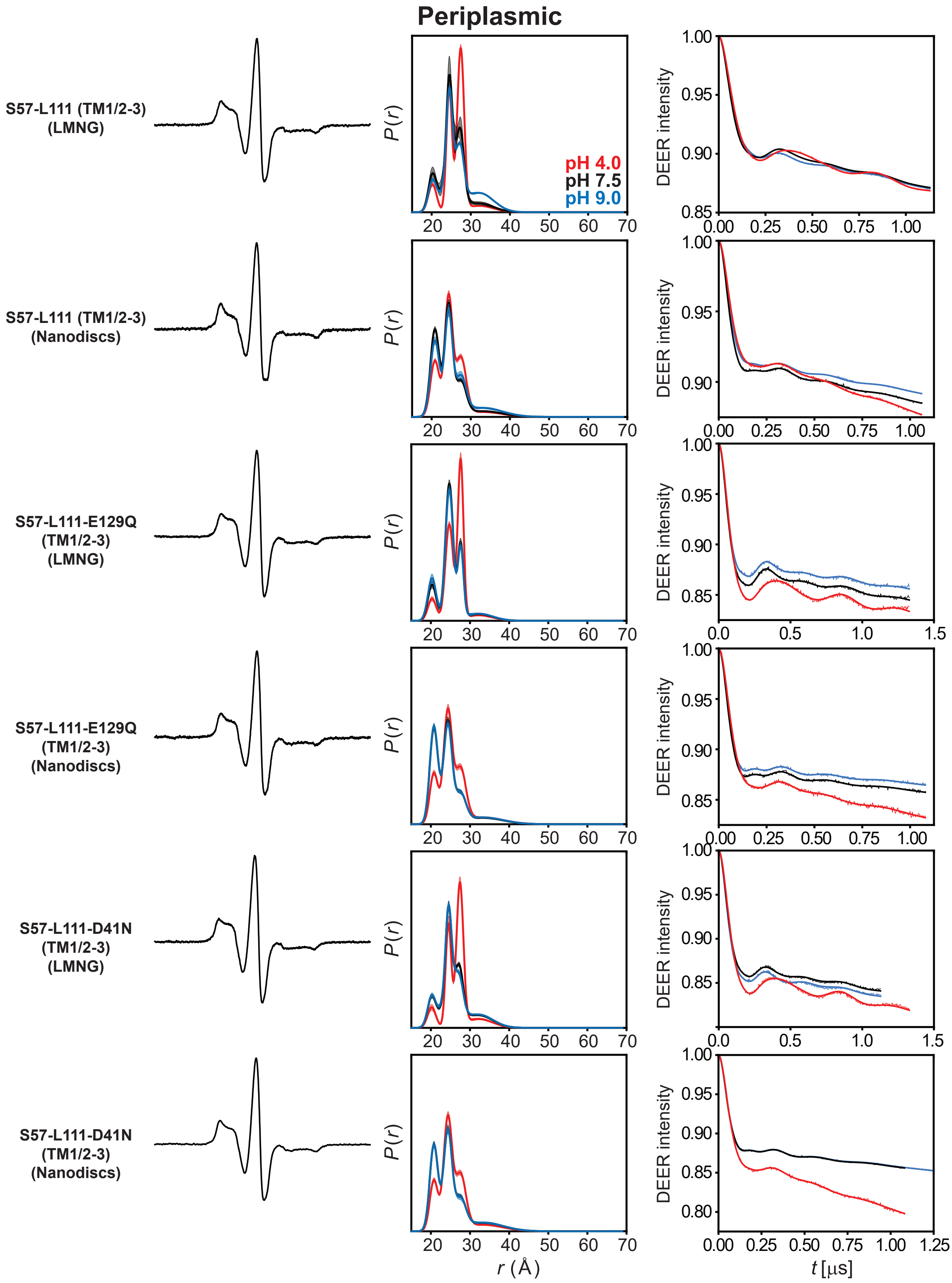

Supplementary Figure 17. DEER data analysis for the periplasmic side. For each mutant, from left to right, CW EPR, distance distributions with confidence bands (2σ) about the best fit lines, and the primary DEER traces along with the fits are shown.

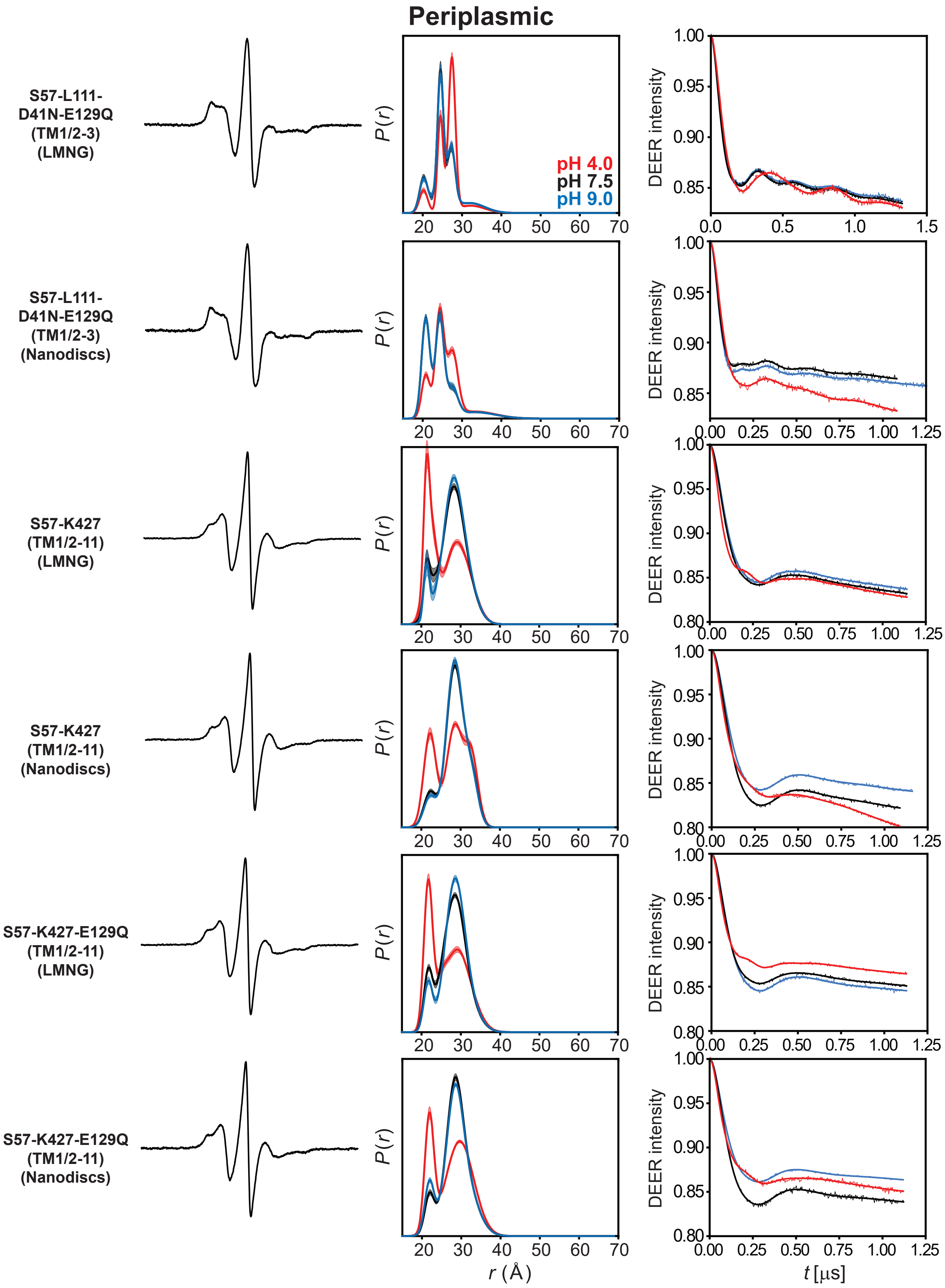

Supplementary Figure 18. DEER data analysis for the periplasmic side. For each mutant, from left to right, CW EPR, distance distributions with confidence bands (2σ) about the best fit lines, and the primary DEER traces along with the fits are shown.

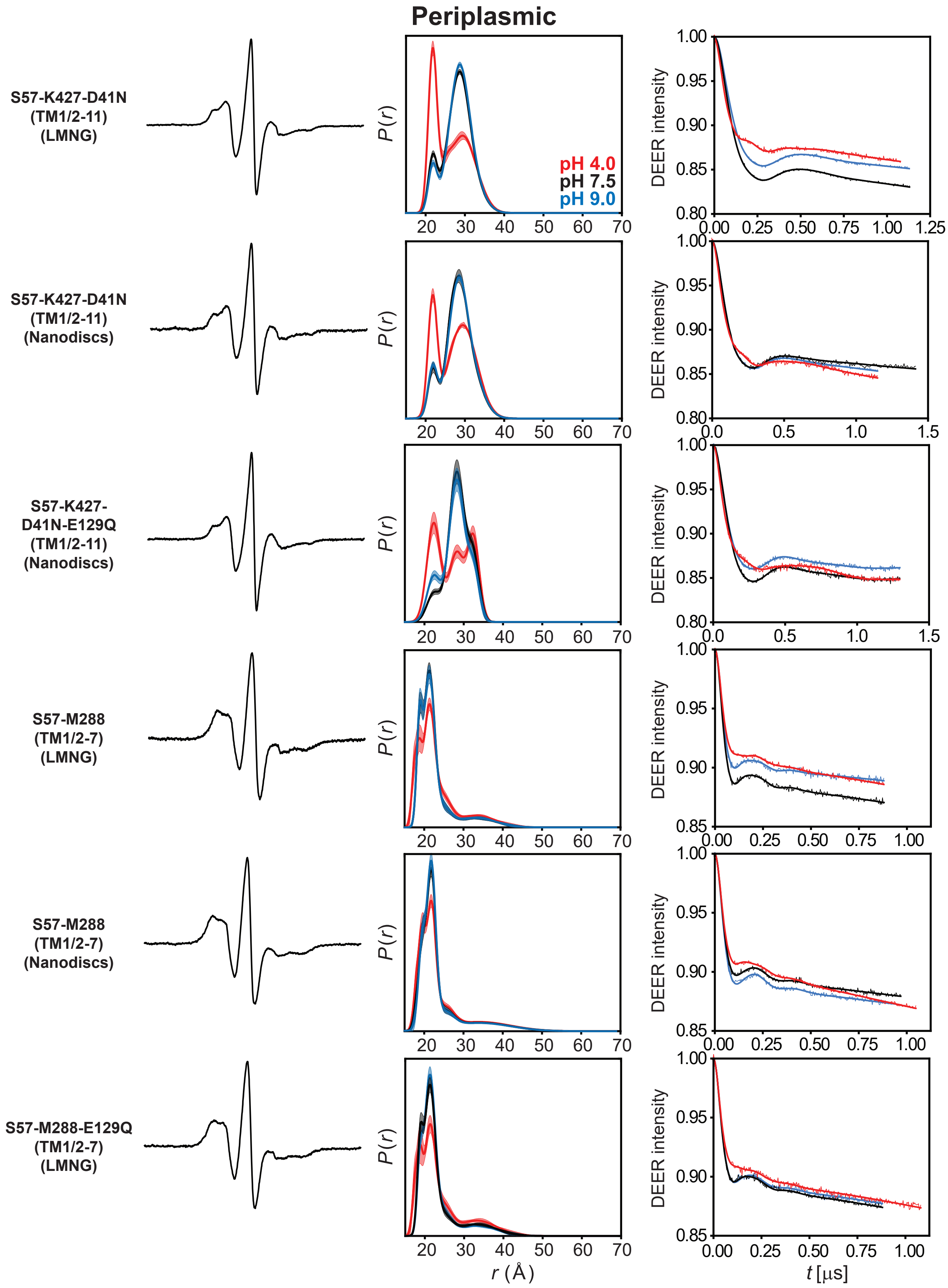

Supplementary Figure 19. DEER data analysis for the periplasmic side. For each mutant, from left to right, CW EPR, distance distributions with confidence bands (2σ) about the best fit lines, and the primary DEER traces along with the fits are shown.

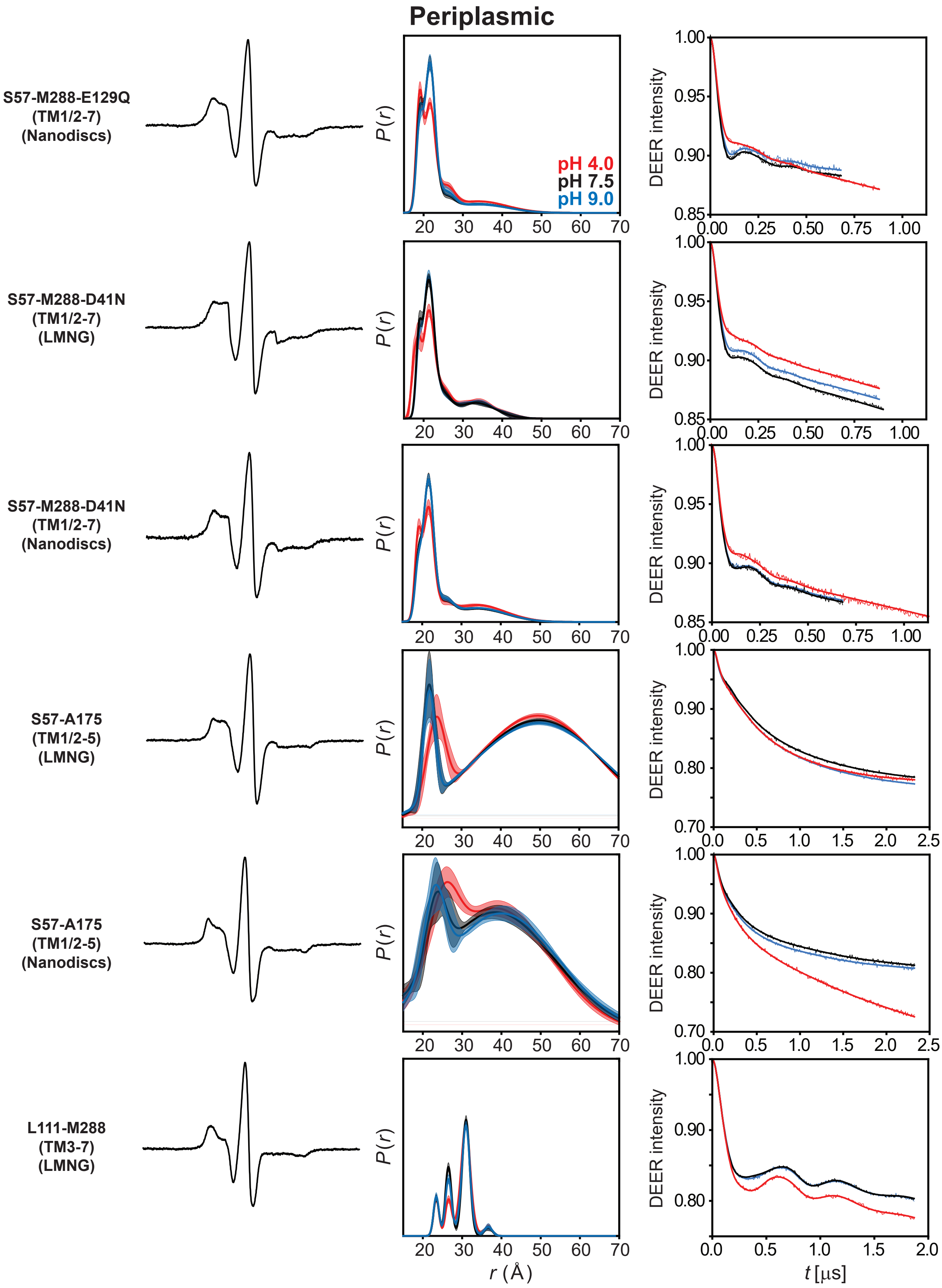

Supplementary Figure 20. DEER data analysis for the periplasmic side. For each mutant, from left to right, CW EPR, distance distributions with confidence bands (2σ) about the best fit lines, and the primary DEER traces along with the fits are shown.

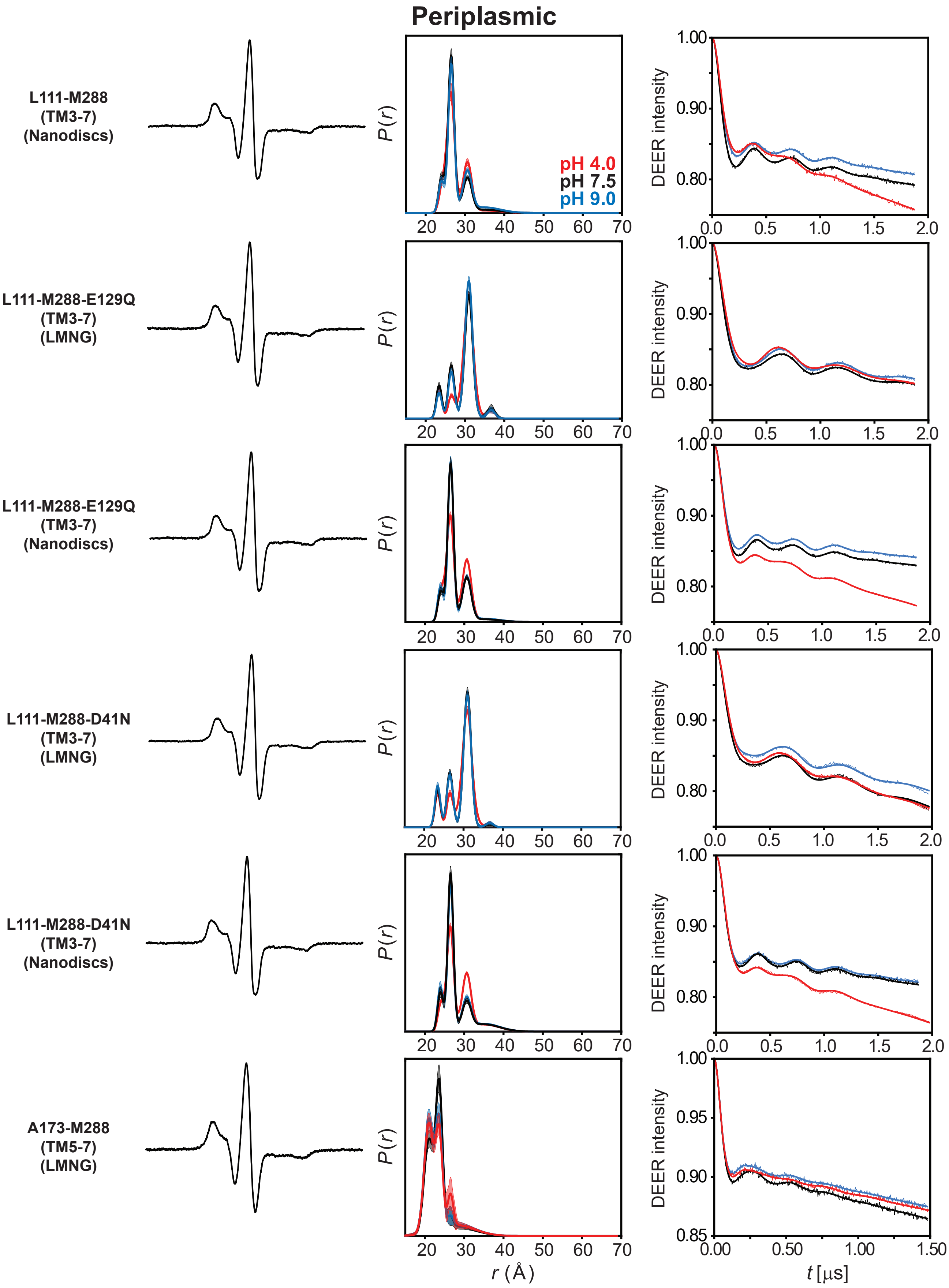

Supplementary Figure 21. DEER data analysis for the periplasmic side. For each mutant, from left to right, CW EPR, distance distributions with confidence bands (2σ) about the best fit lines, and the primary DEER traces along with the fits are shown.

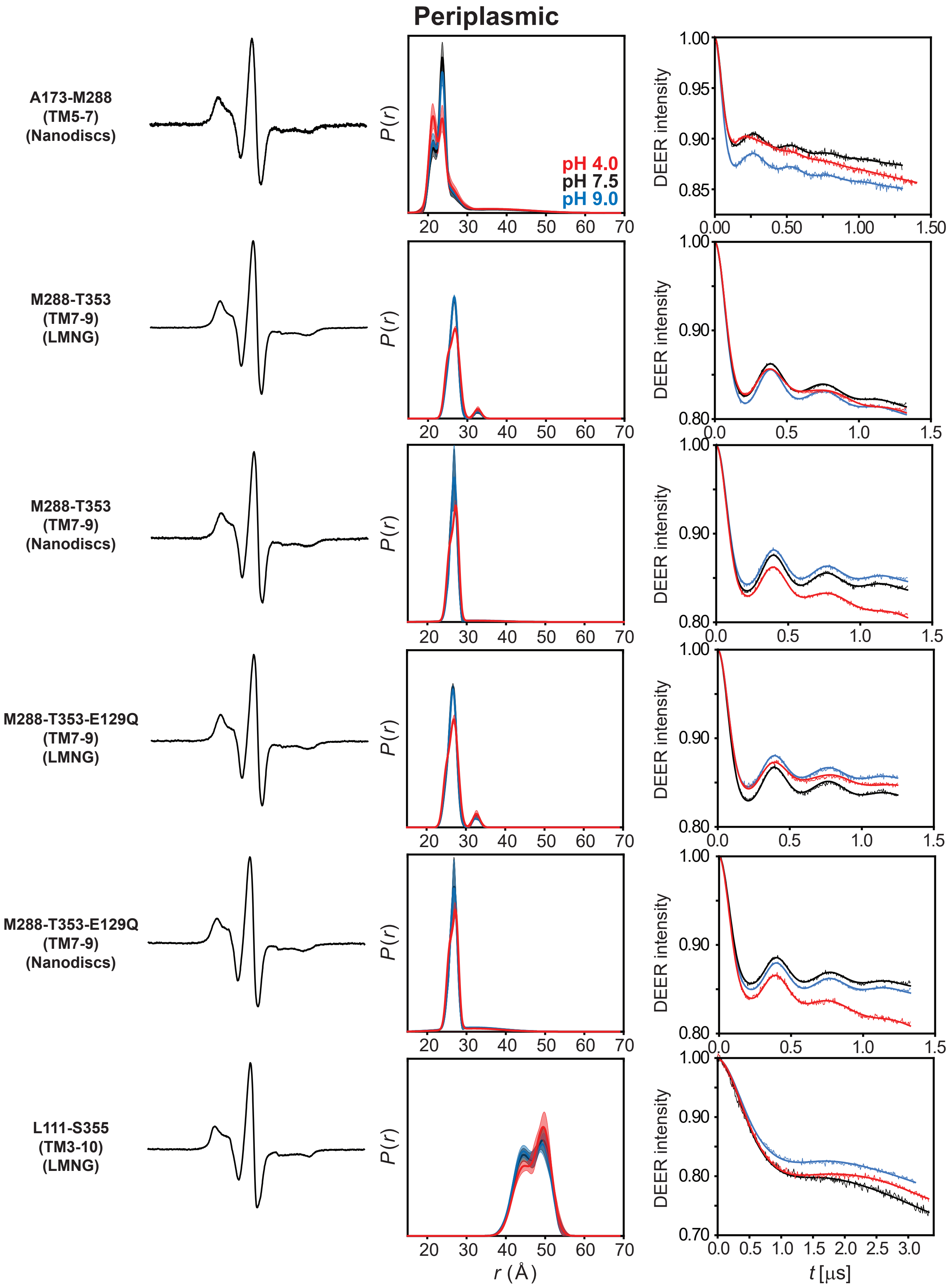

Supplementary Figure 22. DEER data analysis for the periplasmic side. For each mutant, from left to right, CW EPR, distance distributions with confidence bands (2σ) about the best fit lines, and the primary DEER traces along with the fits are shown.

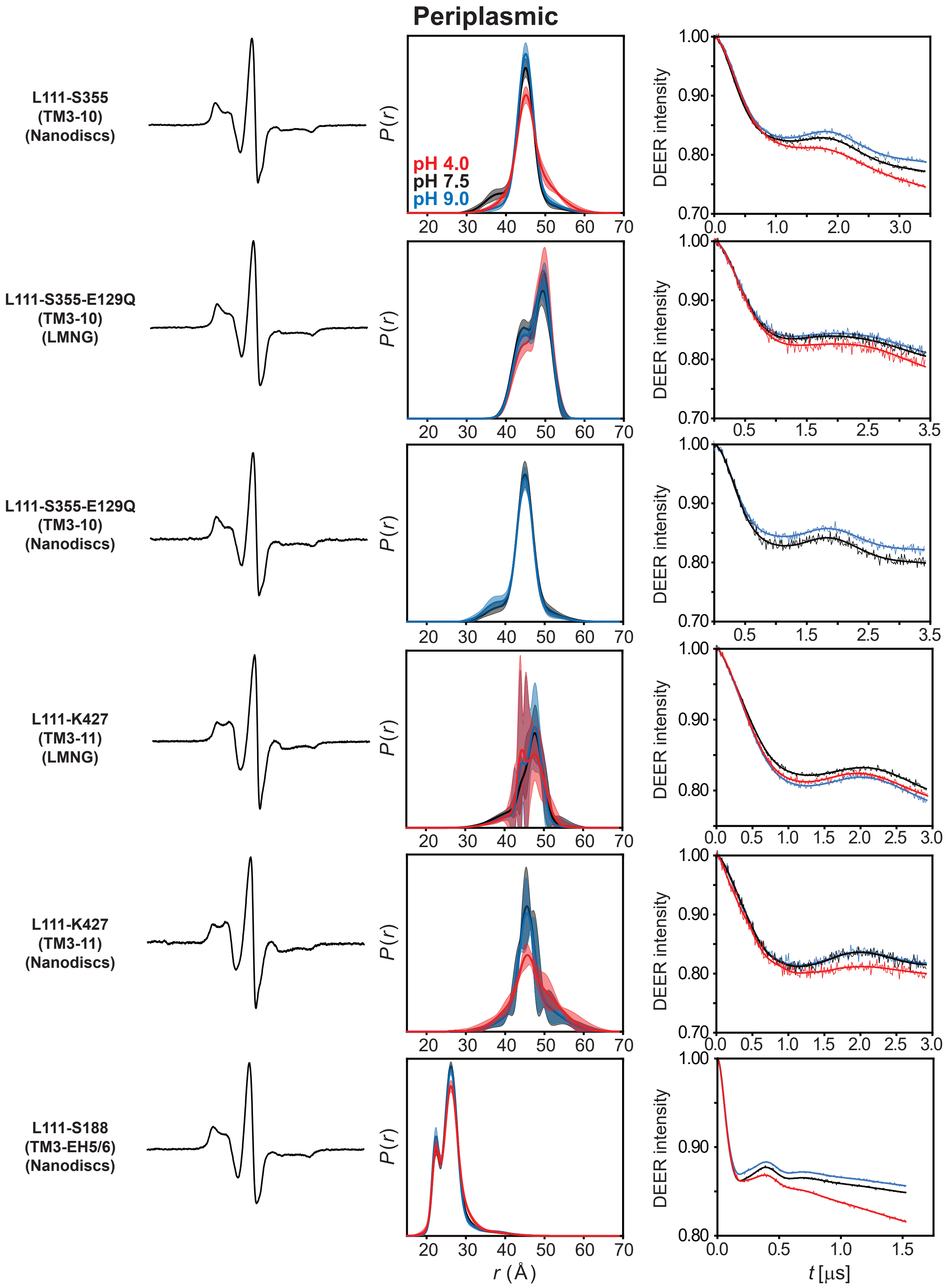

Supplementary Figure 23. DEER data analysis for the periplasmic side. For each mutant, from left to right, CW EPR, distance distributions with confidence bands (2σ) about the best fit lines, and the primary DEER traces along with the fits are shown.

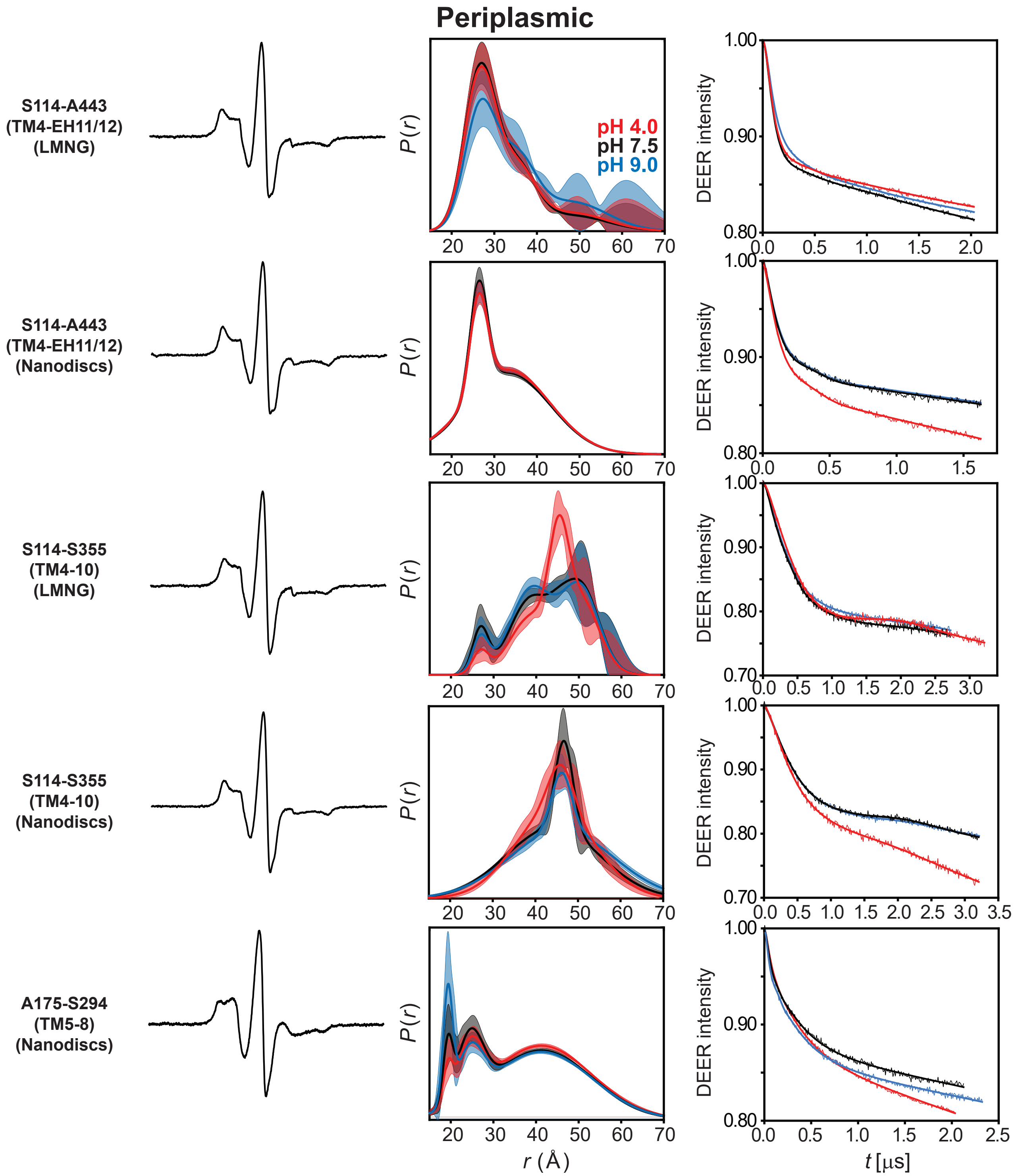

Supplementary Figure 24. DEER data analysis for the periplasmic side. For each mutant, from left to right, CW EPR, distance distributions with confidence bands (2σ) about the best fit lines, and the primary DEER traces along with the fits are shown.

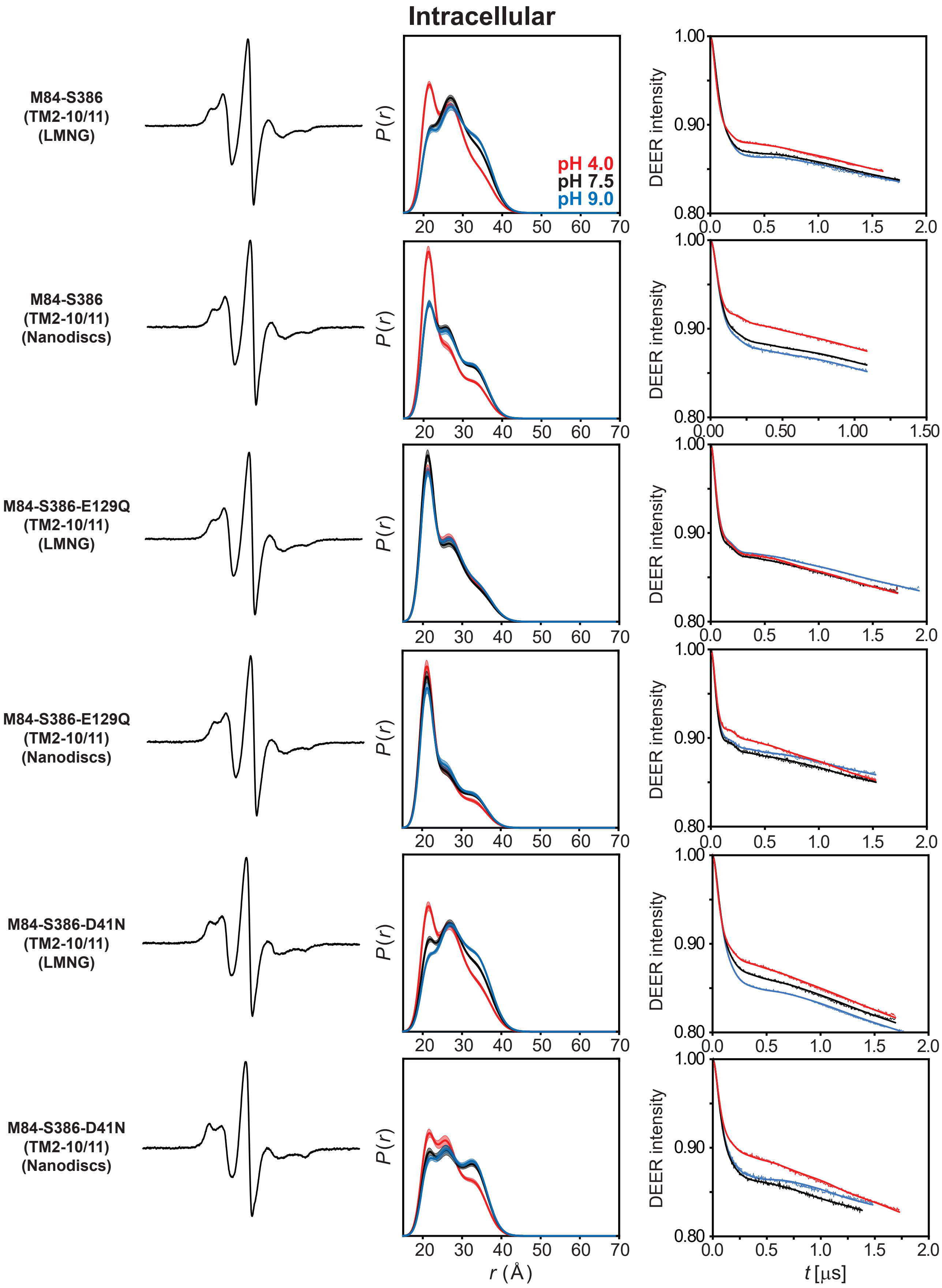

Supplementary Figure 25. DEER data analysis for the intracellular side. For each mutant, from left to right, CW EPR, distance distributions with confidence bands (2σ) about the best fit lines, and the primary DEER traces along with the fits are shown.

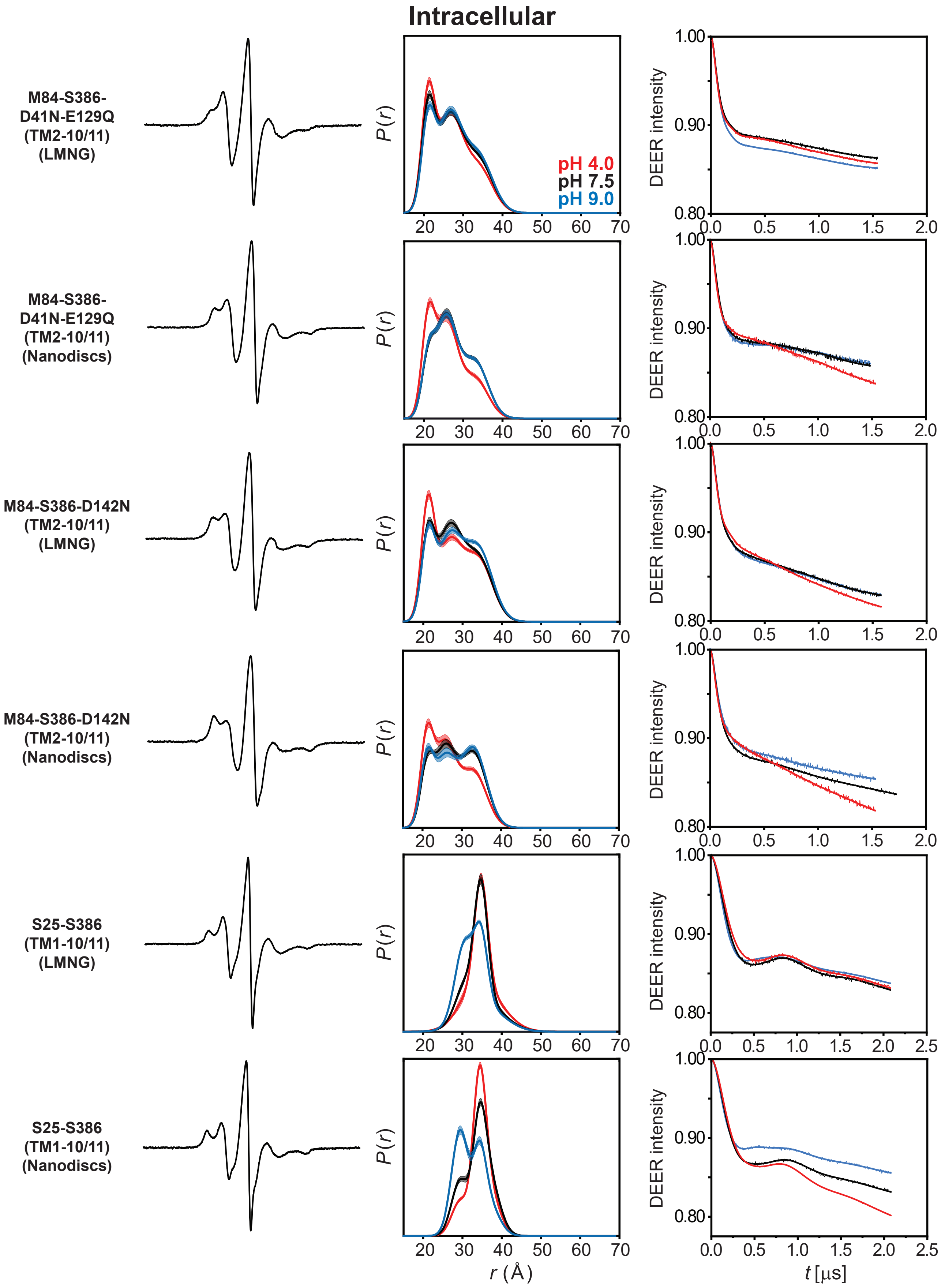

Supplementary Figure 26. DEER data analysis for the intracellular side. For each mutant, from left to right, CW EPR, distance distributions with confidence bands (2σ) about the best fit lines, and the primary DEER traces along with the fits are shown.

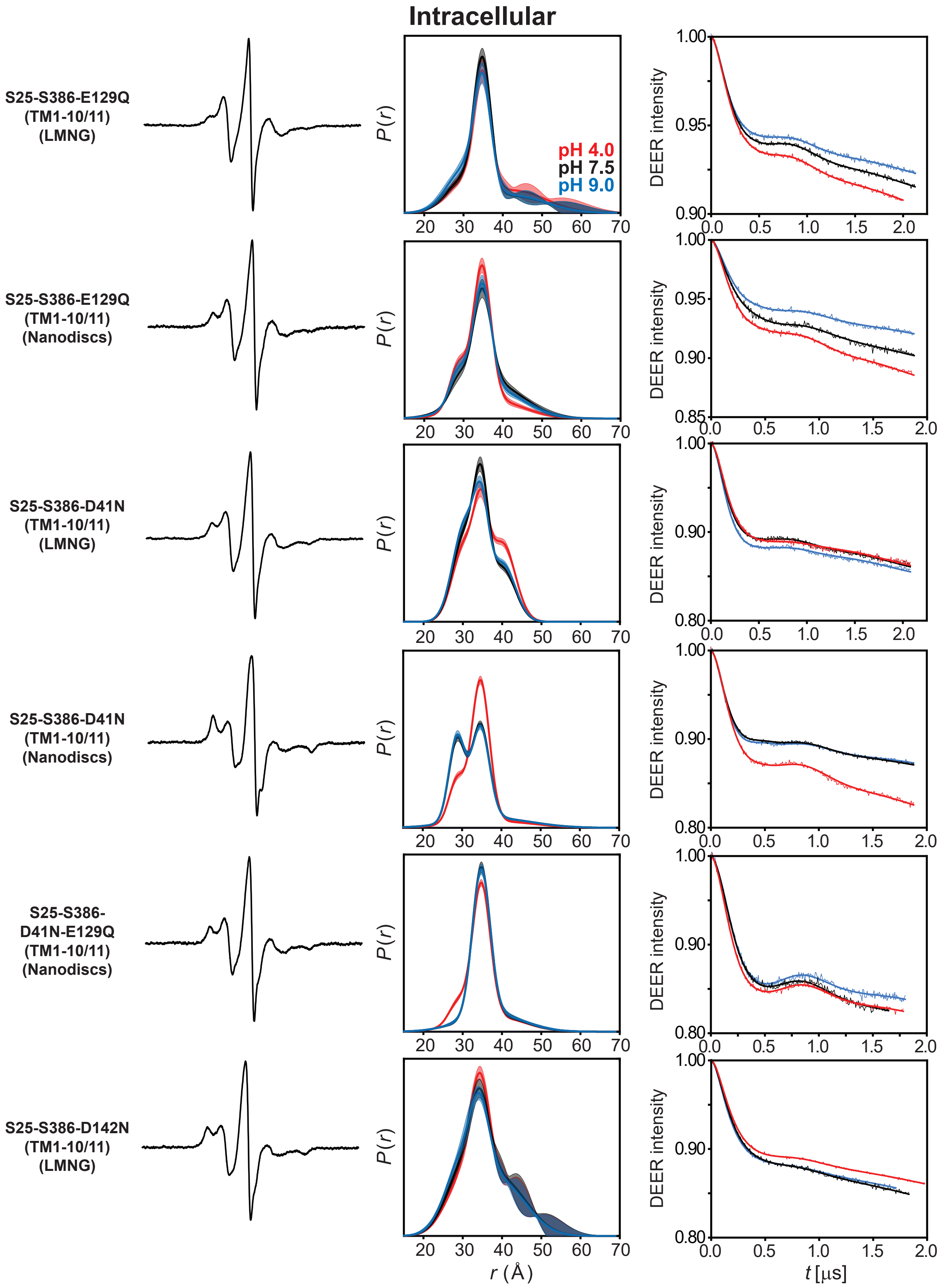

Supplementary Figure 27. DEER data analysis for the intracellular side. For each mutant, from left to right, CW EPR, distance distributions with confidence bands (2σ) about the best fit lines, and the primary DEER traces along with the fits are shown.

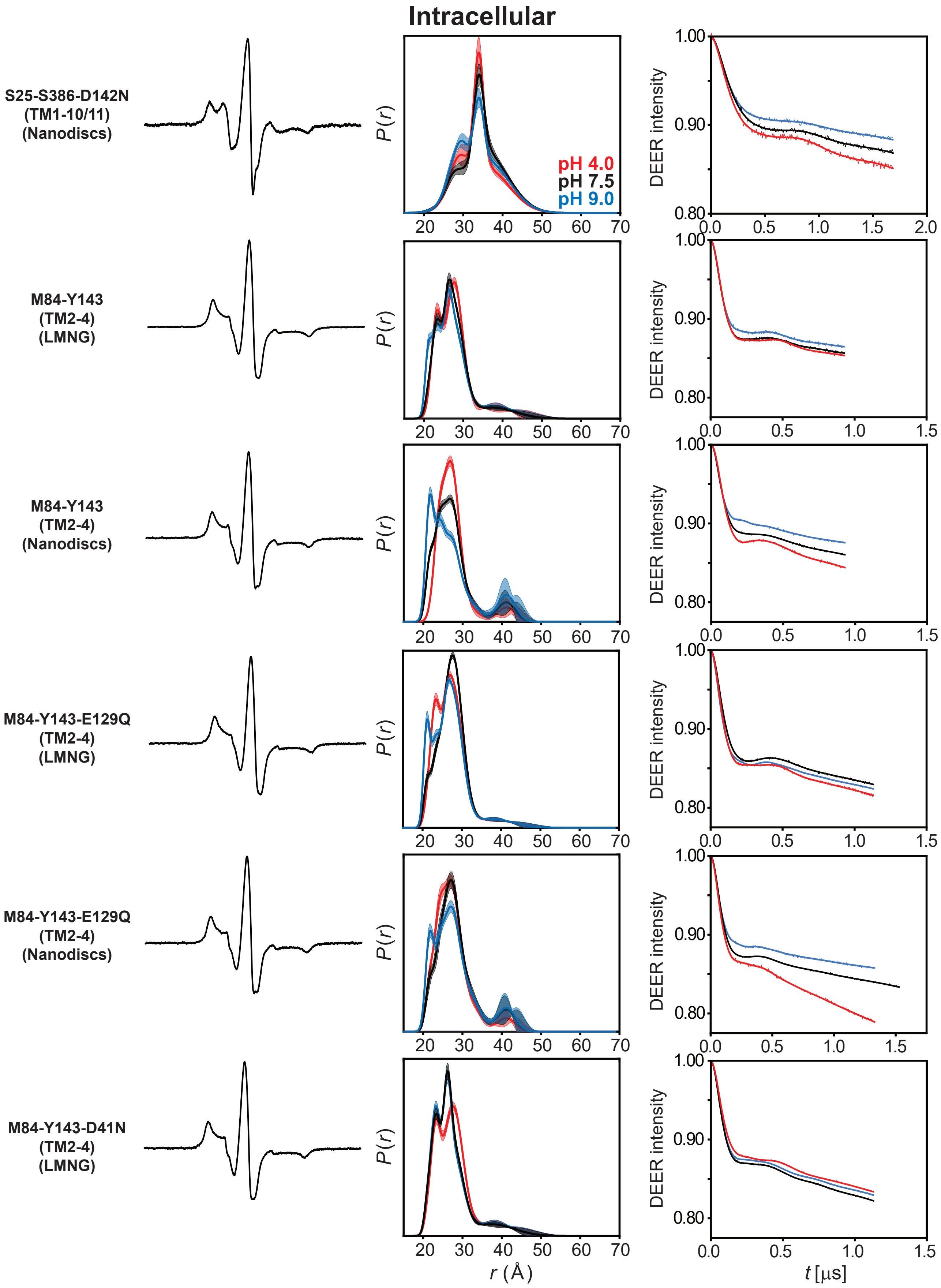

Supplementary Figure 28. DEER data analysis for the intracellular side. For each mutant, from left to right, CW EPR, distance distributions with confidence bands (2σ) about the best fit lines, and the primary DEER traces along with the fits are shown.

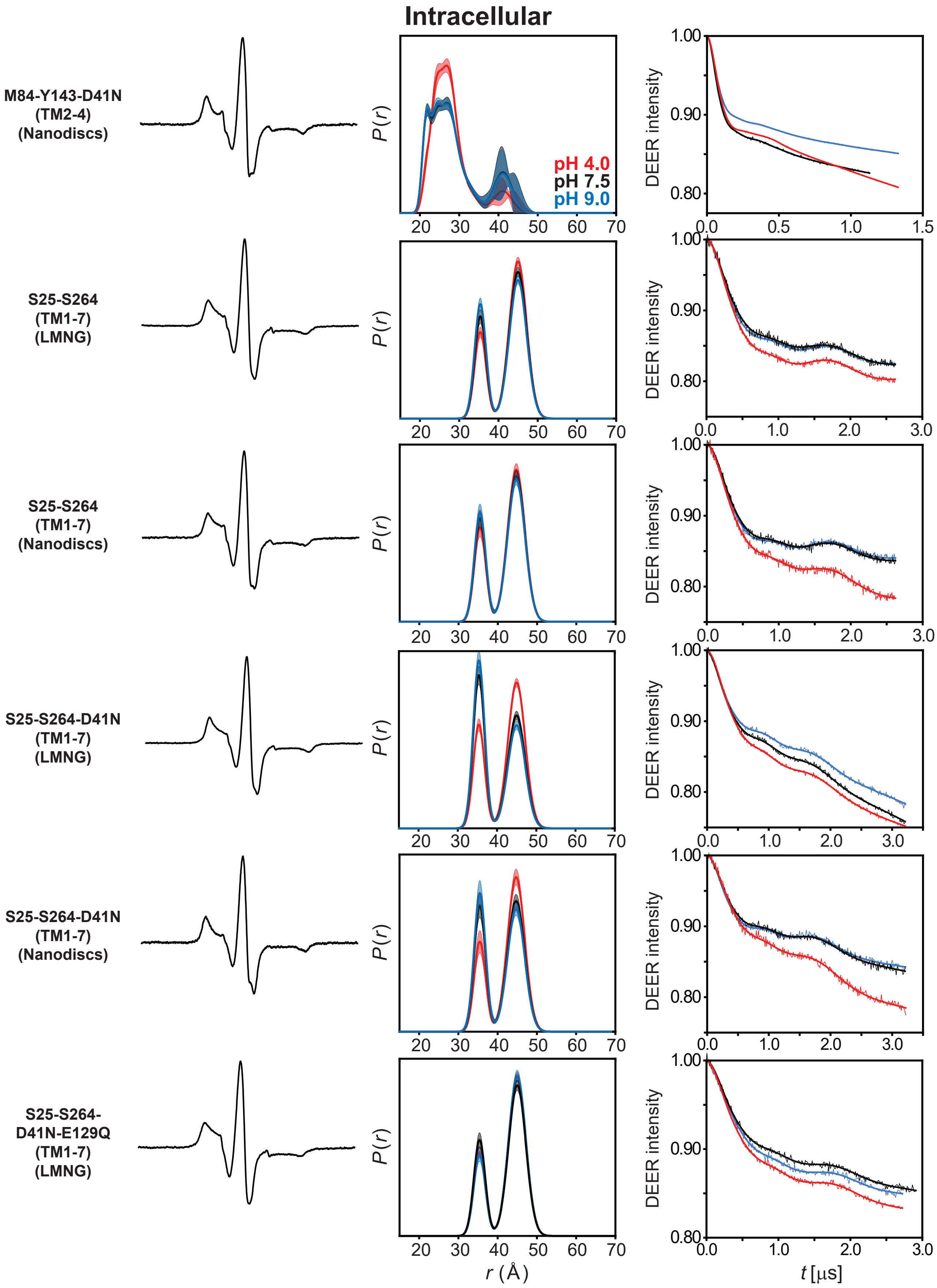

Supplementary Figure 29. DEER data analysis for the intracellular side. For each mutant, from left to right, CW EPR, distance distributions with confidence bands (2σ) about the best fit lines, and the primary DEER traces along with the fits are shown.

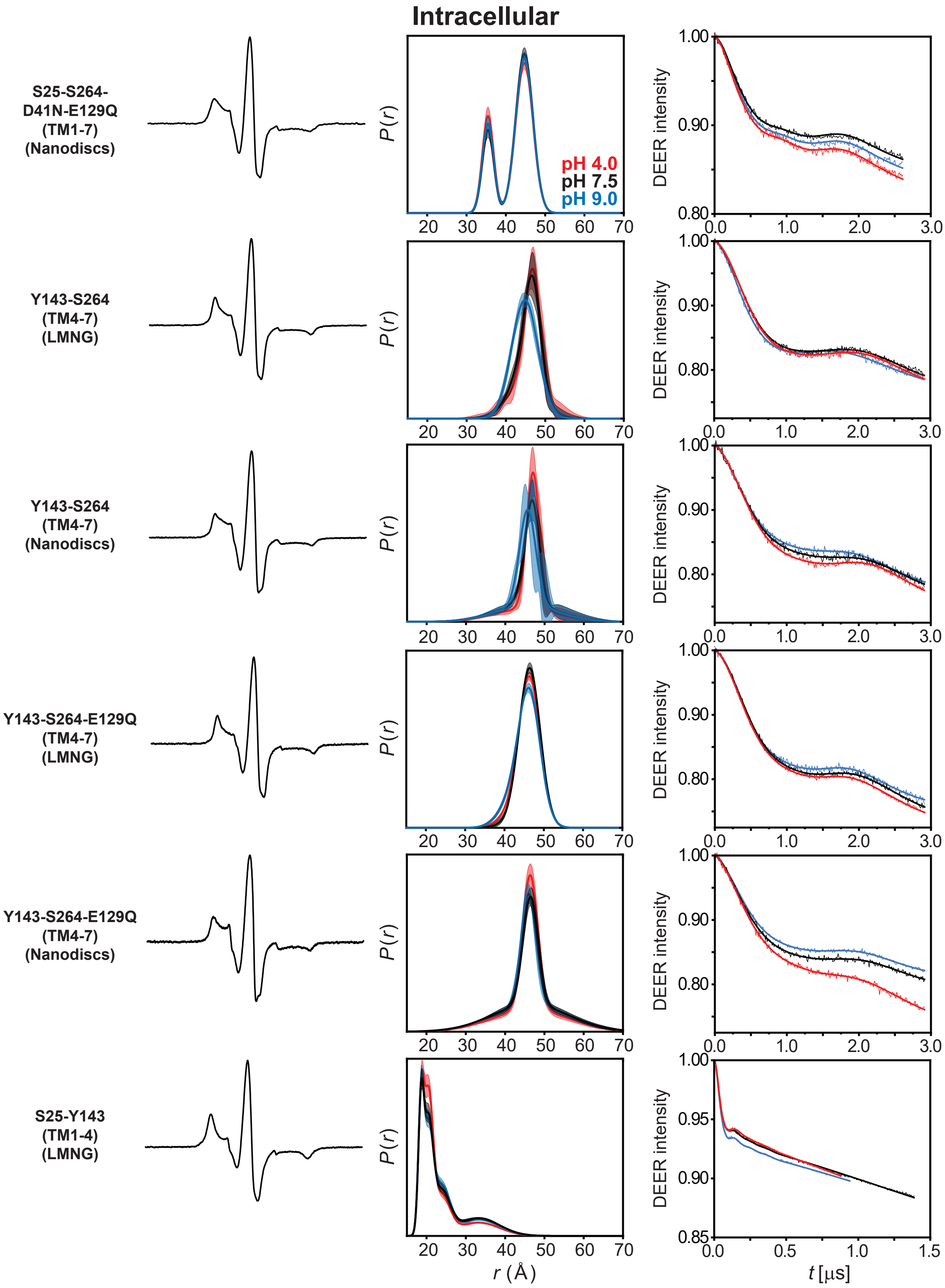

Supplementary Figure 30. DEER data analysis for the intracellular side. For each mutant, from left to right, CW EPR, distance distributions with confidence bands (2σ) about the best fit lines, and the primary DEER traces along with the fits are shown.

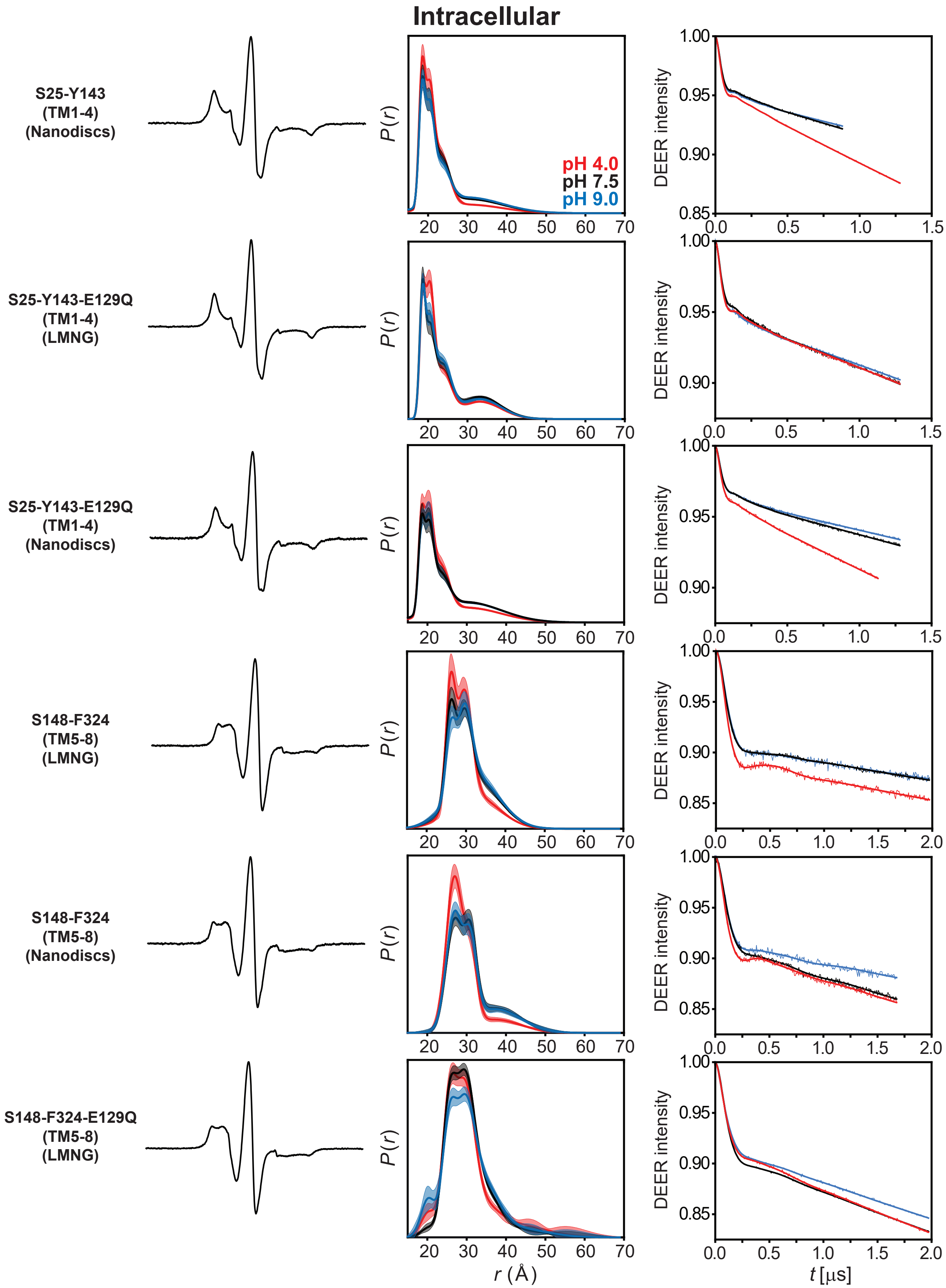

Supplementary Figure 31. DEER data analysis for the intracellular side. For each mutant, from left to right, CW EPR, distance distributions with confidence bands (2σ) about the best fit lines, and the primary DEER traces along with the fits are shown.

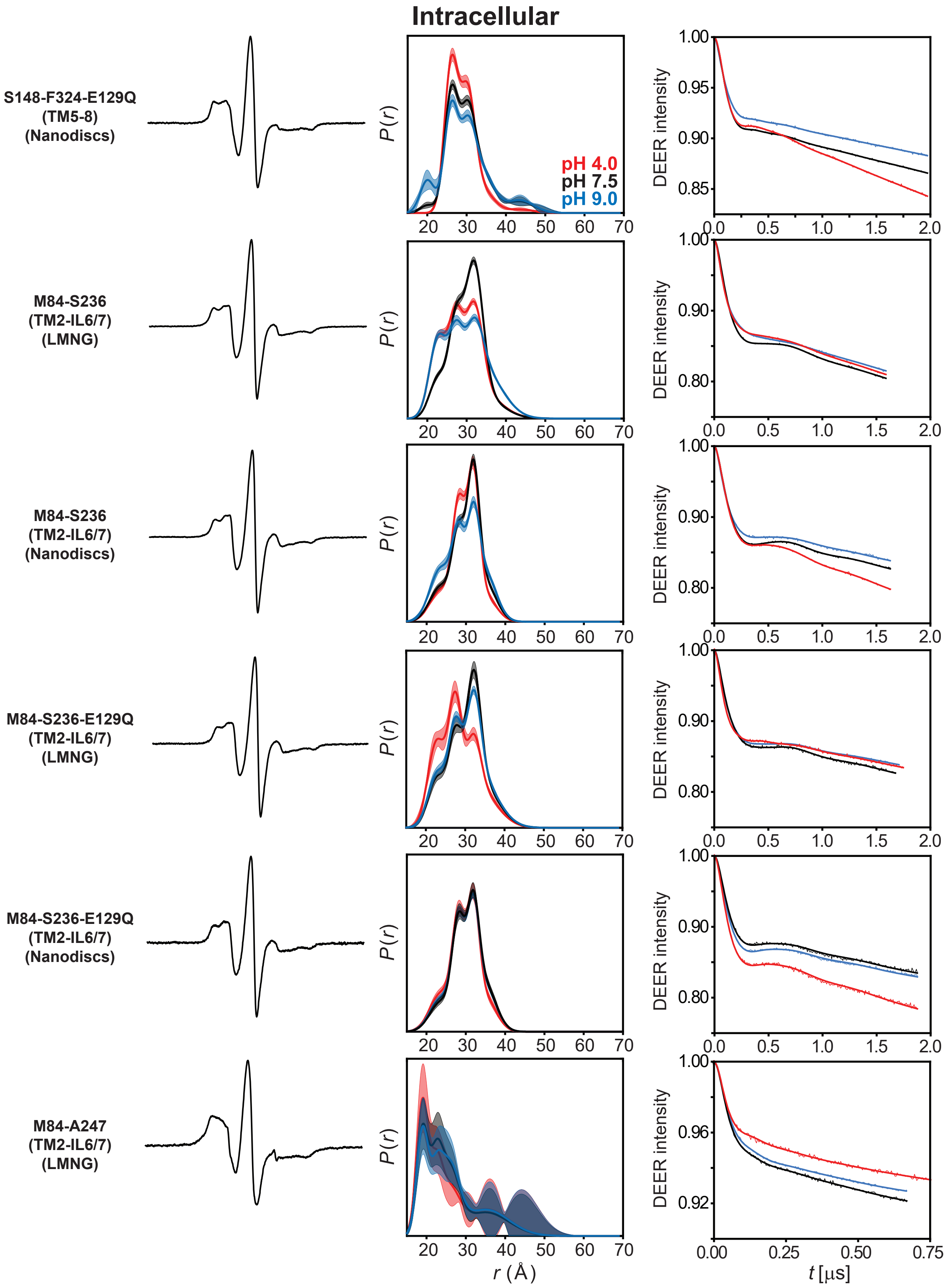

Supplementary Figure 32. DEER data analysis for the intracellular side. For each mutant, from left to right, CW EPR, distance distributions with confidence bands (2σ) about the best fit lines, and the primary DEER traces along with the fits are shown.

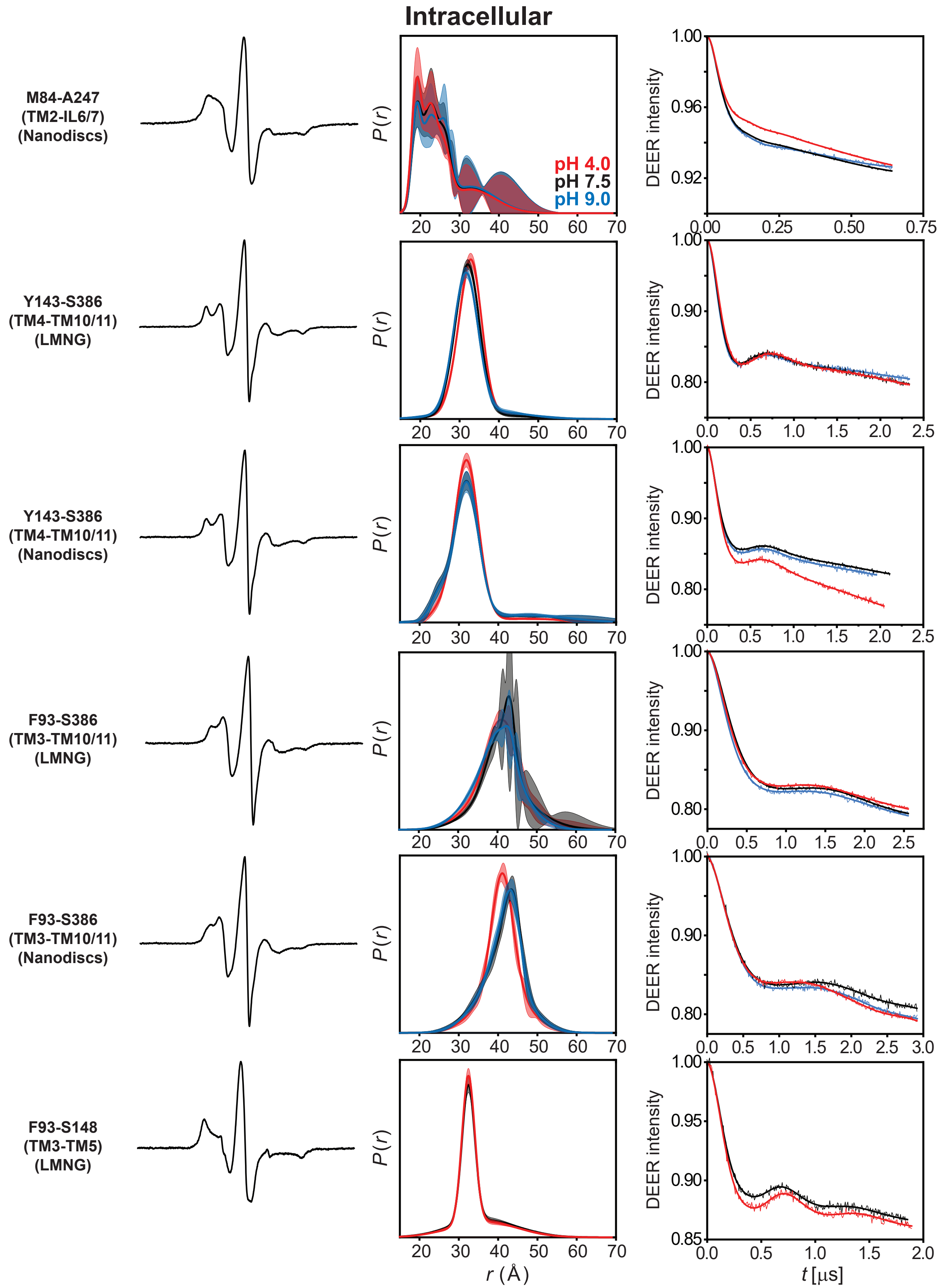

Supplementary Figure 33. DEER data analysis for the intracellular side. For each mutant, from left to right, CW EPR, distance distributions with confidence bands (2σ) about the best fit lines, and the primary DEER traces along with the fits are shown.

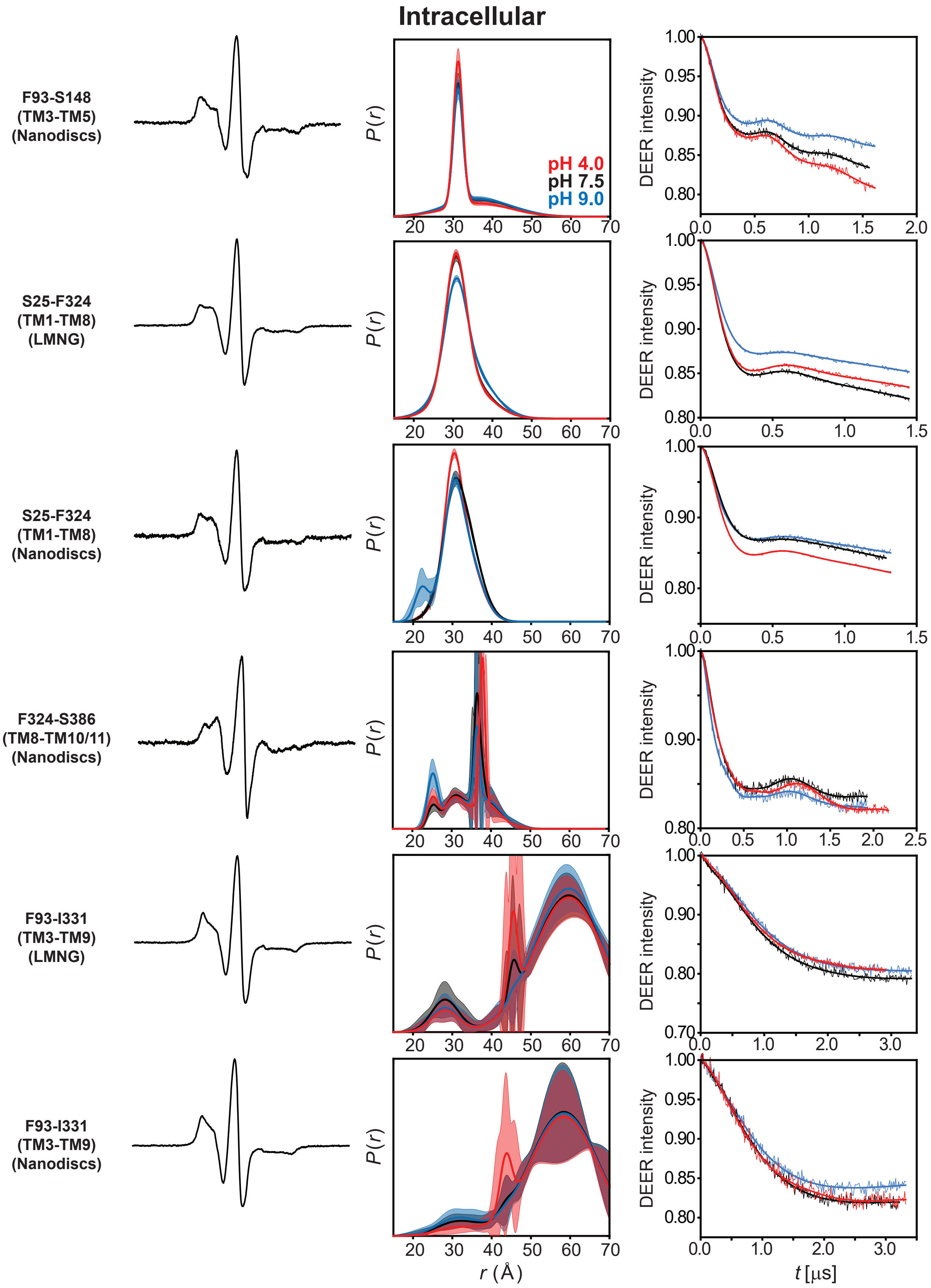

Supplementary Figure 34. DEER data analysis for the intracellular side. For each mutant, from left to right, CW EPR, distance distributions with confidence bands (2σ) about the best fit lines, and the primary DEER traces along with the fits are shown.

Supplementary Table 1. Melting temperatures (T_m) of spin-labeled DEER and protonation-mimetic mutants in LMNG micelles and lipid nanodiscs.

	**T_m (°C) ± Fitting error**	
**Mutant**	**LMNG**	**ND**
CL	58.1±0.1	58.3±0.1
CL-E129Q	57.7±0.1	57.3±0.1
CL-D142N	60.0±0.2	72.8±0.4
CL-D41N	62.2±0.2	58.8±0.2
CL-D41N-E129Q	64.2±0.1	71.3±0.2
M84C-S386C	54.8±0.4	56.6±0.5
M84C-Y143C	58.8±0.1	57.5±0.2
S25C-S386C	55.6±0.1	54.3±0.1
S25C-S264C	58.0±0.2	64.1±0.9
S148C-F324C	58.4±0.3	58.7±0.5
Y143C-S264C	58.4±0.1	71.3±0.3
Y143C-S386C	54.7±0.3	61.8±0.2
S57C-K427C	55.7±0.1	54.8±0.2
S57C-L111C	58.0±0.2	58.1±0.1
L111C-M288C	54.7±0.2	56.1±0.1
S114C-S355C	61.2±0.3	54.9±0.1
F93C-S148C	59.2±0.1	72.1±0.5
S25C-Y143C	58.4±0.1	58.6±0.2
M84C-S236C	58.0±0.1	61.8±0.2
S25C-F324C	58.5±0.1	
M84C-A247C	57.1±0.1	59.8±0.2
F93C-I331C	57.5±0.1	67.7±0.3
F93C-S386C	56.3±0.1	77.3±0.7
S25C-M84C	56.5±0.1	
F324C-S386C		63.8±0.1
S57C-A175C	61.5±0.3	59.9±0.2
S114C-A443C	60.7±0.5	70.6±0.7
L111C-S355C	56.2±0.1	64.6±0.2
A175C-S294C	58.9±0.1	58.8±0.7
M288C-T353C	53.9±0.3	62.9±0.3
A173C-M288C	55.5±0.1	
L111C-K427C		79.0±1.0
L111C-S188C		60.2±0.2

Supplementary Table 2. *p* values determined from a two-tailed independent *t*-test comparing the drug resistance ($Abs_{600\ nm}$) of *Ms*Spns WT with pET19b vector (n_{vector} = 3 for capreomycin and ethidium bromide, n_{vector} = 4 for rifampicin).

**Capreomycin** (*n* = 3, df = 4)			
Concentration (μg/mL)	2 hours	4 hours	6 hours
0	0.0017<br>**	0.0026<br>**	0.0822<br>ns
8.8	<0.0001<br>****	<0.0001<br>****	0.0002<br>***
17.5	<0.0001<br>****	<0.0001<br>****	<0.0001<br>****
52.5	0.0024<br>**	<0.0001<br>****	<0.0001<br>****
70	0.0003<br>***	<0.0001<br>****	<0.0001<br>****
**Rifampicin** (*n* = 3, df = 5)			
Concentration (μg/mL)	2 hours	4 hours	6 hours
0	<0.0001<br>****	0.074<br>ns	0.9008<br>ns
2.5	<0.0001<br>****	<0.0001<br>****	0.0006<br>***
5	<0.0001<br>****	0.0002<br>***	<0.0001<br>****
10	<0.0001<br>****	0.0005<br>***	0.0381<br>*
**Ethidium bromide** (*n* = 3, df = 4)			
Concentration (μg/mL)	2 hours	4 hours	6 hours
0	<0.0001<br>****	<0.0001<br>****	0.0194<br>*
8.8	<0.0001<br>****	0.0006<br>***	0.0010<br>***
17.5	0.0005<br>***	<0.0001<br>****	0.0235<br>*
35	<0.0001<br>****	<0.0001<br>****	0.0192<br>*
52.5	0.0002<br>***	<0.0001<br>****	0.0023<br>**
70	0.0016<br>**	<0.0001<br>****	0.0004<br>***

* *p* value ≤ 0.05
** *p* value ≤ 0.01
*** *p* value ≤ 0.001
**** *p* value ≤ 0.0001
ns: not significant
n: number of technical replicates
df: degrees of freedom

Supplementary Table 3. *p* values determined from a two-tailed independent *t*-test comparing the drug resistance (relative $Abs_{600\ nm}$) of *Ms*Spns WT with pET19b vector (n_{vector} = 3 for capreomycin and ethidium bromide, n_{vector} = 4 for rifampicin).

**Capreomycin** (*n* = 3, df = 4)			
Concentration (µg/mL)	2 hours	4 hours	6 hours
8.8	0.0068<br>**	0.0001<br>****	0.0251<br>*
17.5	0.0159<br>*	<0.0001<br>****	0.0006<br>***
52.5	0.3330<br>ns	0.0005<br>***	0.0001<br>****
70	0.1740<br>ns	0.0006<br>***	<0.0001<br>****
**Rifampicin** (*n* = 3, df = 5)			
Concentration (µg/mL)	2 hours	4 hours	6 hours
2.5	0.4767<br>ns	0.0498<br>*	0.0915<br>ns
5	0.2607<br>ns	0.0039<br>**	0.0002<br>***
10	0.0024<br>**	0.1618<br>ns	0.1327<br>ns
**Ethidium bromide** (*n* = 3, df = 4)			
Concentration (µg/mL)	2 hours	4 hours	6 hours
8.8	0.2389<br>ns	0.1641<br>ns	0.3628<br>ns
17.5	0.9247<br>ns	0.0008<br>***	0.0992<br>ns
35	0.0502<br>ns	0.0005<br>***	0.0353<br>*
52.5	0.0147<br>*	0.0002<br>***	0.0057<br>**
70	0.0029<br>**	0.0061<br>**	0.0023<br>**

* *p* value ≤ 0.05
** *p* value ≤ 0.01
*** *p* value ≤ 0.001
**** *p* value ≤ 0.0001
ns: not significant
n: number of technical replicates
df: degrees of freedom

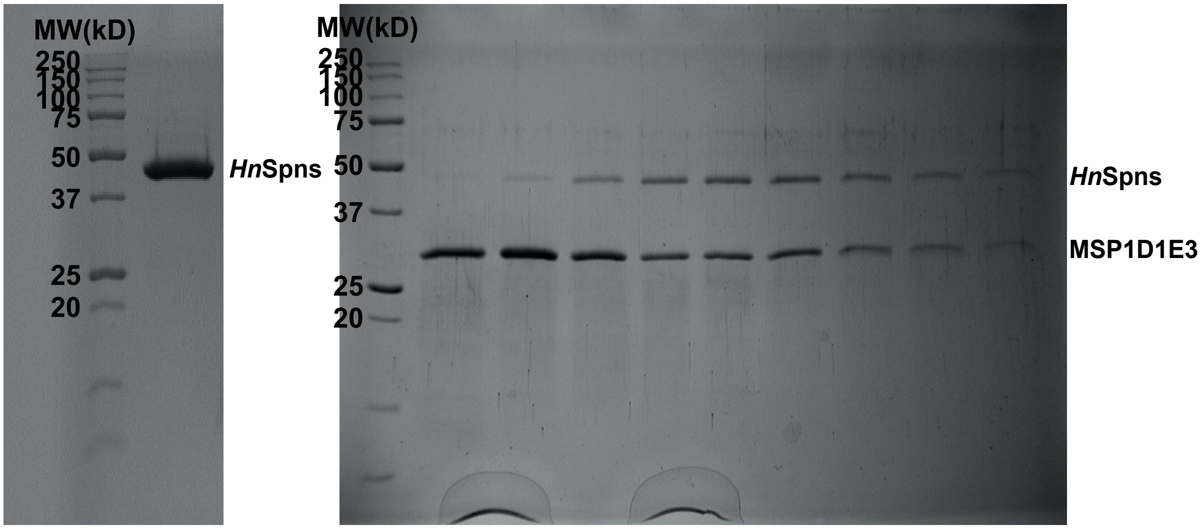

Supplementary Figure 1a

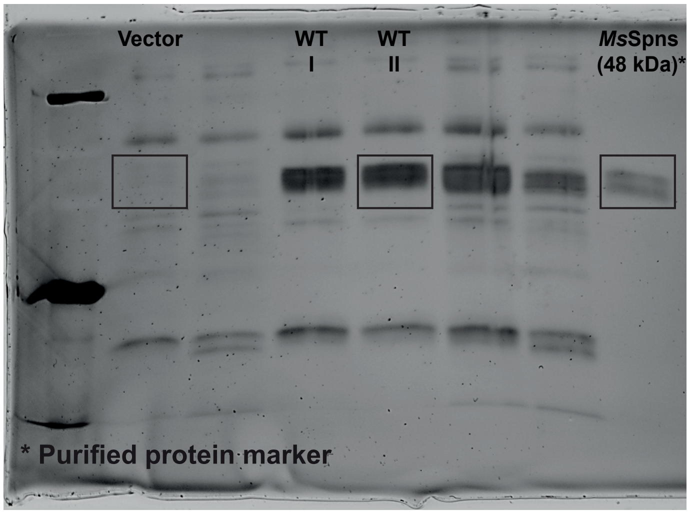

Supplementary Figure 2a
