# Supplementary material for: Proton-driven alternating access in a spinster lipid transporter
Source: Nat Commun. 2022 Sep 2;13:5161. doi: 10.1038/s41467-022-32759-2 (PMC9440201; doi:10.1038/s41467-022-32759-2)
Supplement: Supplementary file 3 — Reporting Summary [file 41467_2022_32759_MOESM3_ESM.pdf]

nature portfolio

Corresponding author(s): Reza Dastvan, Emad Tajkhorshid

Last updated by author(s): Jul 1, 2022

Reporting Summary

Nature Portfolio wishes to improve the reproducibility of the work that we publish. This form provides structure for consistency and transparency in reporting. For further information on Nature Portfolio policies, see our Editorial Policies and the Editorial Policy Checklist.

Statistics

For all statistical analyses, confirm that the following items are present in the figure legend, table legend, main text, or Methods section.

n/a	Confirmed	
☐	☒	The exact sample size (*n*) for each experimental group/condition, given as a discrete number and unit of measurement
☐	☒	A statement on whether measurements were taken from distinct samples or whether the same sample was measured repeatedly
☐	☒	The statistical test(s) used AND whether they are one- or two-sided<br>*Only common tests should be described solely by name; describe more complex techniques in the Methods section.*
☒	☐	A description of all covariates tested
☒	☐	A description of any assumptions or corrections, such as tests of normality and adjustment for multiple comparisons
☐	☒	A full description of the statistical parameters including central tendency (e.g. means) or other basic estimates (e.g. regression coefficient) AND variation (e.g. standard deviation) or associated estimates of uncertainty (e.g. confidence intervals)
☐	☒	For null hypothesis testing, the test statistic (e.g. *F*, *t*, *r*) with confidence intervals, effect sizes, degrees of freedom and *P* value noted<br>*Give P values as exact values whenever suitable.*
☒	☐	For Bayesian analysis, information on the choice of priors and Markov chain Monte Carlo settings
☒	☐	For hierarchical and complex designs, identification of the appropriate level for tests and full reporting of outcomes
☒	☐	Estimates of effect sizes (e.g. Cohen's *d*, Pearson's *r*), indicating how they were calculated

Our web collection on statistics for biologists contains articles on many of the points above.

Software and code

Policy information about availability of computer code

Data collection	NAMD 2.14 was used for metadynamics and reMD simulations.<br>NAMD 3.0 was used for equilibrium MD simulations.
Data analysis	VMD 1.9.3 was used for visualization and analysis of MD simulations.<br>MATLAB R2019b and MMM 2018.2 software package were used for prediction of distance distributions on models and structures.<br>DEER data were analyzed and plotted using DeerA (https://doi.org/10.5281/zenodo.6678447) and OriginPro 2020 (Originlab). The p values for resistance assays were determined by an unpaired t test using Graph Pad.<br>OriginPro 2021 was used for non-linear least squares analyses.<br>MOLEonline web interface (MOLE 2.5, https://mole.upol.cz/online/) was used for the analysis of tunnels in structural models.

For manuscripts utilizing custom algorithms or software that are central to the research but not yet described in published literature, software must be made available to editors and reviewers. We strongly encourage code deposition in a community repository (e.g. GitHub). See the Nature Portfolio guidelines for submitting code & software for further information.

Data

Policy information about availability of data

All manuscripts must include a data availability statement. This statement should provide the following information, where applicable:

- Accession codes, unique identifiers, or web links for publicly available datasets
- A description of any restrictions on data availability
- For clinical datasets or third party data, please ensure that the statement adheres to our policy

The generated data, including those from the DEER experiments, are available in the manuscript or the supplementary materials (Data Appendices). Source data including DEER, MD trajectories, and DeerA software have been deposited to the Zenodo repository maintained by CERN, https://doi.org/10.5281/zenodo.6678447. Other data that support this study are available from the corresponding authors upon reasonable request.

Human research participants

Policy information about studies involving human research participants and Sex and Gender in Research.

Reporting on sex and gender	N/A
Population characteristics	N/A
Recruitment	N/A
Ethics oversight	N/A

Note that full information on the approval of the study protocol must also be provided in the manuscript.

Field-specific reporting

Please select the one below that is the best fit for your research. If you are not sure, read the appropriate sections before making your selection.

☒ Life sciences ☐ Behavioural & social sciences ☐ Ecological, evolutionary & environmental sciences

For a reference copy of the document with all sections, see nature.com/documents/nr-reporting-summary-flat.pdf

Life sciences study design

All studies must disclose on these points even when the disclosure is negative.

Sample size	No statistical methods were used to predetermine sample size. Sample size for different assays and analyses are specified.
Data exclusions	No data excluded.
Replication	At least three independent sets of MD simulations were conducted to investigate: 1) proton-coupled conformational response (four replicates), 2) capturing a stable occluded conformation, and 3) structural refinement of the IF and occluded conformations by incorporating the experimental DEER data. In resistance assays, each data point was performed in triplicate and two biological repeats were performed to successfully establish the reproducibility. For DEER experiments, for sites that prove critical for our conclusion, data were obtained from two biological repeats. All attempts at replication were successful.
Randomization	Experiments were not randomized. N/A
Blinding	The investigators were not blinded to allocation during experiments and outcome assessment. N/A

Reporting for specific materials, systems and methods

We require information from authors about some types of materials, experimental systems and methods used in many studies. Here, indicate whether each material, system or method listed is relevant to your study. If you are not sure if a list item applies to your research, read the appropriate section before selecting a response.

Materials & experimental systems

n/a	Involved in the study
☒	☐ Antibodies
☒	☐ Eukaryotic cell lines
☒	☐ Palaeontology and archaeology
☒	☐ Animals and other organisms
☒	☐ Clinical data
☒	☐ Dual use research of concern

Methods

n/a	Involved in the study
☒	☐ ChIP-seq
☒	☐ Flow cytometry
☒	☐ MRI-based neuroimaging
